# Exploring the challenges to safer prescribing and medication monitoring in prisons: A qualitative study with health care staff

**DOI:** 10.1371/journal.pone.0275907

**Published:** 2022-11-03

**Authors:** Esnath Magola-Makina, Aseel S. Abuzour, Darren M. Ashcroft, James Dunlop, Petra Brown, Richard N. Keers

**Affiliations:** 1 Division of Pharmacy & Optometry, University of Manchester, Manchester, United Kingdom; 2 Patient Safety Research Unit, Greater Manchester Mental Health NHS Foundation Trust, Manchester, United Kingdom; 3 NIHR Greater Manchester Patient Safety Translational Research Centre, University of Manchester, Manchester, United Kingdom; 4 Pharmacy Department, Pennine Care NHS Foundation Trust, Ashton-Under-Lyne, United Kingdom; Public Library of Science, UNITED KINGDOM

## Abstract

**Introduction:**

Research suggests that patients who are prisoners experience greater morbidity, increased health inequalities and frequent preventable harm, compared to the general population. Little is known about the process and influencing factors for safe prescribing in the unique prison environment, which may limit the development efforts to improve the quality of care in prisons. This study aimed to understand the process and challenges associated with prescribing in prisons, explore the causes and impact of these challenges, and explore approaches to improve prescribing safety in prisons.

**Methods:**

Grounded theory informed data collection and analysis of a nominal group discussion by seven participants and semi-structured telephone interviews with twenty prison healthcare staff, including GPs, pharmacists, psychiatrists and nurses.

**Findings:**

The underlying complexity of prescribing in prison settings increased the level of challenge and influenced the safety of this process. Multiple contributors to the challenges of safe prescribing were identified (comprising governance and policy; the prison structure; staff retention, training and skill mix; IT systems and interface; polypharmacy and co-morbidity; tradability and patient behaviour) with overarching constructs of variations in practice/policy and the influence of prison culture. Participants identified measures to address these challenges through multi-disciplinary collaborative working, increased consistency in processes, and the need for more innovation and education/training.

**Conclusions:**

Our study highlighted that healthcare provision in prisons is unique and needs to tailor the care provided to patients without enforcing a model focused on primary, secondary or tertiary care. Participants emphasised a necessary shift in workplace culture and behaviour change to support improvements. The COM-B model of behaviour change may be effectively applied to develop interventions in organisations that have in-depth understanding of their own unique challenges.

## Introduction

Patients residing in prison are a unique and particularly vulnerable group. Research from China and the US [1], indicates that prisoners typically experience greater morbidity and health needs than the general population [2] including a higher prevalence of mental health problems, infectious diseases including COVID-19, and other long term conditions [3–6]. Despite these needs, studies in Europe, the US, UK, Australia and Canada suggest prisoners may suffer health inequalities such as reduced access to, and suboptimal treatment for mental health support, psycho-social interventions and over the counter medicines [7–10]. The prison population has been documented as having higher levels of socio-economic deprivation; in particular poorer levels of education, more likely to have been in foster care and more likely to have a history of substance misuse [11].

The need to focus on the quality of care provided to prisoners has been emphasised in recent years in the United Kingdom (UK) following high levels of self-harm, suicide rates, substance abuse, violence and deaths in custody or shortly after release [12]. Overcrowding [13], diversion of prescribed medication for illicit use, inappropriate polypharmacy (where multiple medications are prescribed and not optimised) [14], inadequate staff levels [15], reduced access to healthcare [9] and organisational culture [16] have been identified as challenges to the quality of care. Much of the harm resulting from adverse events (an unwanted/unintended health care event which may arise due to medication error, poor safety culture, poor transfer of information or inadequate staff training) in prison is thought to be preventable [17]. As the single biggest intervention used in the management of health problems, research that focusses on the quality and safety of medication use may be timely. This requires detailed understanding of the patient who is a prisoner, and the prison as a setting for healthcare provision to understand the impact they have on prescribing.

Understanding and improving the safe use of medicines in health care services is currently an international priority, led by the World Health Organisations (WHO) Third Global Patient Safety Challenge ‘Medication Without Harm’ [18]. Extensive research has identified the scale and causes of medication safety problems in primary and secondary care, and in particular for prescribing and medicines administration which are the most commonly affected medication use stages [19–21]. Interventional studies using patient safety toolkits or information technology (such as the pharmacist-led PINCER trial) are being used in these settings now seek to address these challenges [22–24], however there has been very little comparative research conducted in prisons. Indeed, recent reviews of medication errors, adverse drug events and preventable harm across health care settings failed to identify any studies originating from this setting [25, 26]. Limited research to date has focused on those with mental health problems, revealing high rates of potentially inappropriate prescribing, inadequate management of specific illnesses and problems on entry to custody [27–29]. Whilst UK quality/safety standards and guidance exist relating to medication use in prison [14, 30, 31], they are either generic frameworks for practice or focus on issues such as medicines diversion without considering more broadly the challenges and influences on safe medication use practices. Bartlett *et al*. [10] describe some of the challenges and influences on psycho-active medication prescribed in prison, citing illicit drug use, the adjustment to prison life, or limited access to alternative (non-drug) treatments. They further recognise the need for further research, to explore for example the links between illicit drug use and the organisation of the prison.

It is therefore important to explore the healthcare staff experiences of medicines management, and in particular prescribing and medication monitoring, in prisons. Medicines management is defined as ‘The clinical, cost-effective and safe use of medicines to ensure patients get the maximum benefit from the medicines they need, while at the same time, minimising potential harm [32]. Examination of recent studies exploring the causes of medication incidents in mental health hospitals [33] reveals that the unique characteristics of the prison setting and its population warrant tailored solutions to safety challenges. The high turnover rate of the prison population, varying security requirements, the presence of multiple commissioned providers from the NHS, private and third sector organisations and involvement of multiple health professionals (some of which may not be based in prisons) may influence the quality and safety of prescribing [34–38]. In addition, the professionals who may prescribe in prisons (such as prison general practitioners [GPs], psychiatrists, substance misuse specialists and non-medical prescribing pharmacists and nurses), review the appropriateness of prescribing, audit therapeutic drug monitoring (such as clinical pharmacists) or inform local policy (such as clinicians/managers with a role in patient safety and policy makers) determine the quality and safety of prescribing. This complexity suggests that prescribing safety improvements may need to be designed to flexibly accommodate local needs. This study therefore aimed to characterise and understand the processes and challenges to prescribing in prisons, explore the causes and impact of these challenges, and identify potential approaches to improve prescribing safety in prisons. Specifically, focus groups were designed to capture harms, as part of wider work to develop indicators of potentially hazardous prescribing (reported here [39]), and interviews (where in depth discussion of risks and benefits) aimed to identify areas of good practice. Prescribing safety indicators describe statements of potentially hazardous prescribing practices that may place patients at risk of harm, and have been utilised previously as part of a pharmacist-led intervention successfully implemented in general (or community) practice and secondary care [40].

## Methods

A two-stage qualitative study design combining the nominal group discussion (NGD) and semi-structured interviews was iteratively used in the study to produce findings that are close to and representative of the prison setting.

### Stage 1: Nominal group discussion (NGD)

#### Design

A focus group using NGD was first used to identify and prioritise the nature of prescribing challenges in prisons, as well as the harms resulting from them. NGD is a structured discussion in response to a nominal question, where individually-generated expert participant contributions are shared, before being discussed by the whole group [41, 42]. Since little was about the real-world nature of prescribing challenges in prisons the structured NGD approach was better suited to identify and prioritise research aims. This inductive approach then ultimately informed the nuanced questions and structure of an interview guide for stage 2.

#### Recruitment

Targeted recruitment of expert healthcare professionals (prison General Practitioners (GPs), psychiatrists, pharmacists, non-medical prescribers, clinicians/managers with a role in medicines management/safety, nurses, policy makers, and substance misuse specialist with a current role in prison settings) defined as having at least 3 years’ experience working in secure environments and an interest in medication safety, took place through invitations shared on social media and within professional networks. Those who expressed an interest in the study were provided with a participant information sheet before completing a consent form.

#### Data collection

Each participant was asked to respond to the nominal question "What medication-related errors/harms or examples of hazardous prescribing are most likely to occur in the prison setting and what is their potential severity?” (S1 File). Following an initial period of silently generating answers to the question, responses were shared in round-robin fashion [which helped reduce bias], before being explored further by all, in an in-depth group discussion that incorporated the wider nature of these prescribing challenges. Finally participants were then asked to individually select and rank the responses in order of priority. The NGD took place in September 2018 on University premises and lasted a total of 3 hours. The NGD was audio-recorded and transcribed verbatim by an institution-approved professional transcribing service. University Research Ethics (Ref: 2018-4516-6805) and National Research Committee (Ref: 2018–211) were granted for the study. Participants were offered a £50 gift voucher for taking part.

### Stage 2: Semi structured interviews

#### Design

Following the NGD, individual in-depth telephone interviews were conducted once with a range of prison health care staff, and were designed to explore in more detail the nature, causes of and interventions to improve prescribing safety challenges in prisons. Individual interviews offered open-ended questions for in-depth exploration of themes raised in the NGD. A semi-structured interview guide (S2 File) was developed using findings from the NGD and a review of the literature.

#### Recruitment

Experienced healthcare professionals (as above from stage 1) from varied prison category settings (S3 File) across England and Wales, were purposively recruited through social media advertisement and professional networks. Those who expressed an interest in the study were provided with an information sheet before written consent to participate was recorded. Participants were offered a £20 gift voucher for taking part.

#### Data collection

Interviews were conducted over the telephone, between October 2019—July 2020, and ranged in duration from 35 to 60 minutes. Questions focussed on participants’ views of the processes of and influences on prescribing practice in prisons, and the nature and contributors to prescribing challenges (S2 File). Any current interventions used to improve the safety of prescribing in prisons were also explored where appropriate. All transcripts were digitally audio-recorded and transcribed verbatim by an institution-approved professional transcribing service. NHS Health Research Authority (Ref: 19/NW/0265) and National Research Committee (Ref: 2019–146) approvals were granted for this phase of the study.

#### Data analysis

Data analysis began during the NGD where EM and RNK grouped participant responses into themes. An anonymised transcript of the discussion was analysed in NVivo 10^®^ (QSR International), using inductive thematic analysis to derive data-driven themes through grounded theory [43]. Transcript analysis was led by EM. Themes interpreted from the transcript were independently reviewed for consensus by RNK and then discussed with the wider research team. A summary of themes was set these themes were then used to generate the interview guide for stage 2.

A constructivist grounded theory approach was used to analyse stage 2 interview data to gain a deeper understanding of prescribing in prisons. The data collected guided analysis, informed themes that were created and influenced subsequent interviews. As is consistent with grounded theory, recruitment was continuous throughout rounds of interviews, however, participant criteria remained the same. NVivo 11^®^ (QSR International) was used as a data management tool while inductive thematic analysis of all transcripts was performed independently by EM in an iterative process [43, 44]. EM listened to interview recordings, making notes on printed transcripts to highlight and code words and quotes of importance. These were then organised into initial themes describing challenges. As more transcripts were coded, comparisons were made between the data and themes were reviewed and refined. AA independently coded half of the interview transcripts and RNK read 25%, before both contributing to the design of the analytical framework. This framework was then applied to all transcripts and further discussion was used to achieve consensus of themes.

Given the interconnectivity of stages 1 and 2 of the study, findings were combined for presentation in the results section.

## Findings

### Participants

Seven prison healthcare professionals participated in the stage 1 NGD including a prison GP, a forensic psychiatrist, four pharmacists and a mental health nurse. This was achieved following targeted recruitment, in which 23 healthcare professionals were invited to participate, 19 expressed an interest in participating, 9 were available to meet at the specified time and date, and seven attended. Where interested participants were unable to attend the nominal group discussion or roles were not represented, invitations to participate in interviews were offered later.

Twenty experienced healthcare professionals (two psychiatrists, one forensic psychiatrist, ten pharmacists, six prison GPs, and one nurse working as a clinician, senior medicines management staff, and substance misuse specialist) of which 16 were prescribers (referred to as prescriber participants) and 4 were non-prescribers (referred to as non-prescriber participants, specifically 3 pharmacists working in medicines optimisation roles and 1 nurse, who was Head of Healthcare) were interviewed by telephone as part of stage 2 of the study. One interview participant had previously taken part in the NGD. The NGD and semi-structured interview participants were healthcare professionals from varied backgrounds working in remand or convicted prisons housing Category A-D male prisoners. The nature of their roles meant most participants worked as employees or locums at between 1 to 6 prison sites each. Three participants however, performed roles which included oversight of over 40 secure sites, as part of their role.

To achieve representation of roles, explore themes first identified in the nominal group discussion, and ensure data saturation, a larger sample size was used. Data saturation for the main themes identified was achieved after fifteen interviews. While no new themes were identified after fifteen interviews, interviews 18–20 provided interesting perspectives on the early impact of the COVID-19 pandemic.

### Key themes

Qualitative data collection from the NGD and semi-structured interviews aimed to identify and explore prescribing challenges and their perceived harms in prison. Themes from the NGD were ranked by participants from most to least important as follows: prescribing specific drugs affecting the central nervous system, inappropriate medicines usage (such as prescribing out of license to accommodate prison regime medicine administration times or accommodate restrictions in opioid prescribing), challenges caused by the system, prisoner behaviours, challenges caused by the process of prescribing, practitioner behaviours. The semi-structured interviews also aimed to provide deeper understanding of the prescribing process and why prescriber challenges occurred, alongside exploration of potential remedial interventions.

Three key themes emerged from the data: (1) prescribing in prisons which sets the scene and provides greater understanding of the prison context and prisoner (2) contributors to prescribing challenges in prison healthcare; where in depth exploration of causes is reported and (3) measures to address prescribing/monitoring challenges. Each section reports findings by key theme.

#### Theme 1: Prescribing in prison

The first key theme to emerge from the data described was prescribing in the prison context. Two subthemes emerged from within this: prescribing for patients who are prisoners; and the impact of prescribing in the prison context.

All participants reported that unique challenges were presented by prescribing in the prison context. In this study, the prison context refers to the interacting structural, social and cultural characteristics forming the prison setting. In addition to the role played by prescribers, healthcare staff and patients, prescribing was also influenced to varying degrees by prison officers and non-healthcare staff at sites, particularly where they provided prescribers with information on prisoner health and medication behaviours.

*Prescribing for patients who are prisoners*. Participants unequivocally shared that having patients who were also prisoners required additional considerations that were less common or absent in general practice. In particular, participants reported a higher incidence of incomplete drug histories and non-registration with a GP, when patients were prisoners. Other considerations included patient consultations with a prison officer present or via a hatch for patients in isolation. [On reception, generally a drug history and medicines reconciliation process is completed to facilitate accurate prescribing during appointments, before patients attend the medicines administration hatch to receive supplies.] Similarly to general practice settings in deprived areas, mental health problems, socio-medical/psycho social needs, illicit drug use were prevalent in prison. Prescribing processes varied depending on initial or repeat medication supplies, the availability of accurate medical/drug histories and how effectively prison formularies or clinical guidelines could be applied.

“*I think one of the things that we always look at is a good history–is there a good history for diagnosis*, *is there a good history for why are they on this medication*? *And can we get that history*…?.” *SSI R7*, *Consultant Psychiatrist*

Prescriber participants reported using observations of patient demeanour and/or physical functionality from non-medical staff to inform prescribing decisions. Like in general practice, these observational or physical cues would assist the prescriber to diagnose and/or identify drug seeking behaviours. Unique to prison, was the fact that some case observations included reports from prison officers’ observations on the wings or gym. Participants reported this as particularly important for high risk medications (opioids, psychotropics or CNS depressants), where patients kept their own medications on wings (In possession/IP), and where the risk of prescribed medicines being misused or traded was high. Prison officer observations also influenced whether analgesia was prescribed to patients reporting pain.

“*We do have an agreement with the prison and we [*..*] send forms to the wing*: *functional assessments*. *We send the form to the wing and we ask them (prison officers) to just mark if they’ve ever seen the person doing this*, *walking up and down the stairs*, *that type of thing*, *playing pool*. *And then we use that to help us make our judgement*. *We also use that with the gym as well; so if the person is going there lifting heavy weights*.” *SSI R8*, *Pharmacist (prescriber)*

“*I would always ask prison officers about how a person had been and what was going on*. *They would be involved in an MDT kind of situation for some of the complex case reviews*” *SSI R18 Nurse (non-prescriber)*

Typically, all participants reported that patients were predisposed to complex socio-medical and psycho-social needs, a high incidence of mental health problems and a history of substance misuse (legal and/or illicit). As seen in the general population, this demographic background was sometimes coupled with physical health needs such as diabetes, epilepsy, infectious disease, geriatric syndrome (commonly defined as including pressure ulcers, incontinence, falls, functional decline and delirium [45]) and poor dental health, and further influenced prescriber and patient behaviours. Patient behaviours are reported on further in Section 2 of the findings.

“… *the population’s ageing* … *I think at this point*, *this is the highest number of patients with comorbidities or complex issues that you might see in a prison setting*. *So actually*, *physical health is becoming just as important or pertinent as mental health and substance misuse in prisons*.” *SSI R8*, *Pharmacist (prescriber)*

*Impact of prescribing in the prison context*. NGD participants emphasised that the overall increased complexity of prisoner healthcare needs over the general population led to incidents of medication or monitoring errors, overdose (intentional or accidental), poor documentation, inappropriate prescribing or delayed/omitted doses. Based on their experiences, all participants reported that prescribing and monitoring challenges increased overall risk of toxicity, intoxication, diversion, violence/bullying, suicide, self-harm and death.

“*[I see] inappropriate dosage regimens leading to missed medications*.” *NGD R3 Pharmacist prescriber*

“*Poor documentation of intoxication events leading to missed opportunity to review and stop medication*.” *NGD R5*, *General Practitioner*

Prescribing in prison was perceived on the whole as prescriber-led, and less patient-centred than in general practice. Some participants expressed apparent disparities was when prescribing within the constraints of prison processes and systems (for example, prescribing medicines off-licence to accommodate prison regimes for administration, lack of MDT team meetings). Prescribers’ perceptions of how prescribed medication may be used/misused by patients in prison, were perceived to contribute to health inequalities, prescriber apathy, and poor patient safety culture. As described by this psychiatrist, organisational culture perceived that prisoners were not entitled to the same level of care as the general population: he felt strongly that this prescribing approach was a punitive.

“*I suppose the other big bugbear of mine is—‘Just because they’re [patients] in prison they’re not entitled to the same care as they are in the community’*…. *we’re not police officers…*. *our role is to appropriately diagnose and treat what’s in front of us*. *…*.*But you don’t automatically say*, *because I’m in prison I’m not going to prescribe this and this to you because it’s abused*, *you might trade it*.” *SSI R7*, *Consultant Psychiatrist*

Inter/intra-professional conflict resulted within prison healthcare teams or between on-site clinicians and external providers because of unclear lines of responsibility for prescribing/drug monitoring or differences in the application of prescribing guidelines and thresholds. For example, the GP below was unhappy with having responsibility for monitoring renal function (includes estimated glomerular filtration rate, eGFR), for drugs prescribed by psychiatry.

“*Every time I have a word with a psychiatrist saying*, *‘If you want to prescribe olanzapine*, *you need to monitor it*.*’ [They respond] ‘Oh well I don’t understand eGFRs’*. …. *the irony is that I can’t prescribe it because I’m not qualified but I have to do all the risky bit and monitor it*.” *SSI R1*, *General Practitioner*

This typically arose where psychiatrists worked directly for prisons and therefore they (psychiatrists rather than the GPs) completed all mental health prescribing, in line with local arrangements. Elsewhere, GPs were able to prescribe repeat supplies of these medicines only after a psychiatrist had initiated prescribing, and authorised GPs to prescribing through a shared-care protocol.

#### Theme 2 –Contributors to prescribing challenges in prison

**Fig 1:**
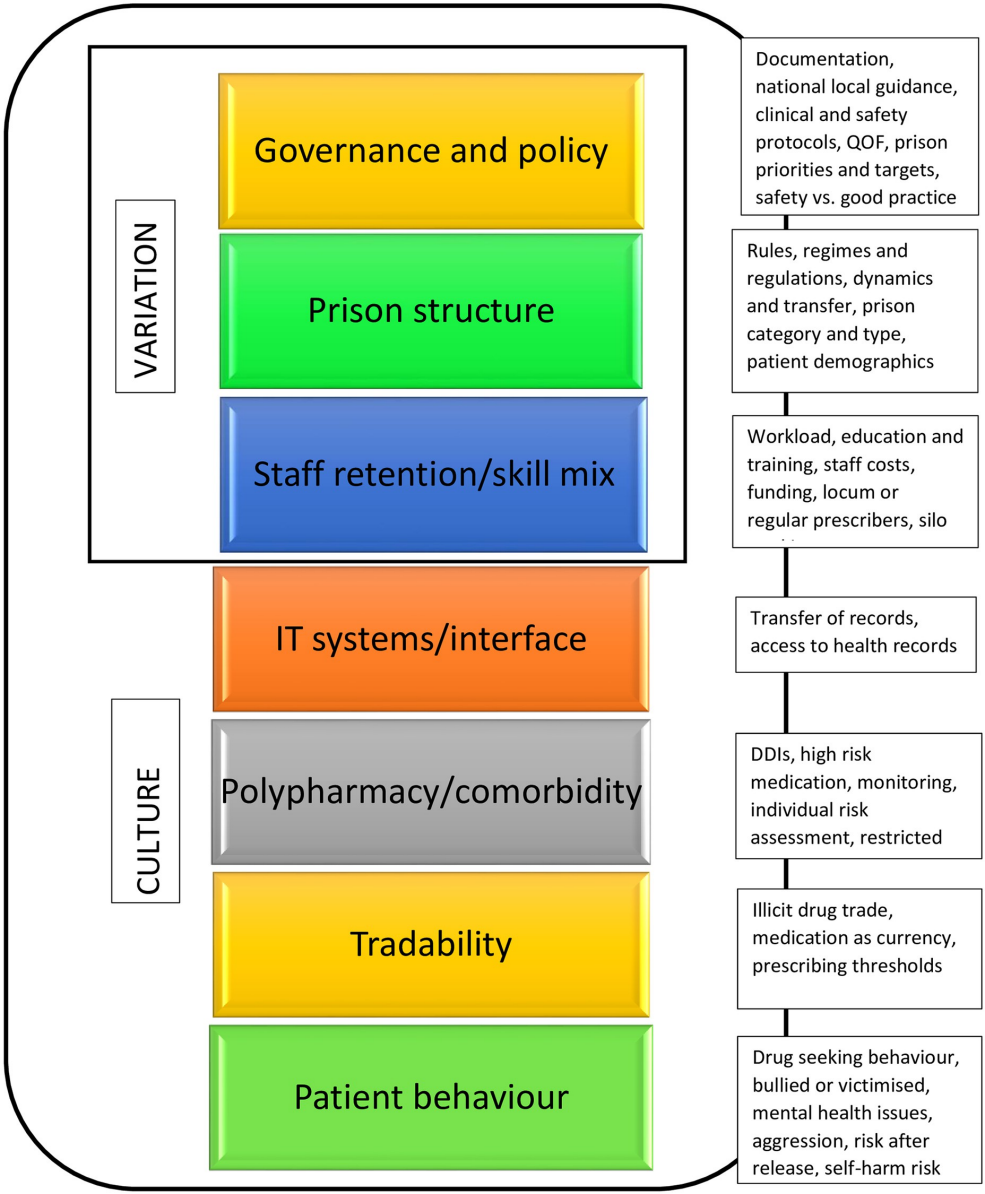
Contributors to prescribing challenges and medication safety.

Within this theme, seven subthemes emerged from the data: governance and policy; the prison structure; staff retention, training and skill mix; IT systems and interface; polypharmacy and co-morbidity; tradability and patient behaviour. Consensus from all participants was that variation (in practice or policy) and culture were the overarching constructs affecting most of these subthemes, as shown in Fig 1.

#### Governance and policy

Eight participants reported how clinical governance structures were absent within their prisons and those with experience of general practice reported that this was a feature more common to prison healthcare. Often, arrangements for healthcare provision appeared to involve a greater combination of NHS or private healthcare providers, clinical commission groups and subcontractors than in general practice. Often these providers worked from different protocols, clinical and risk management guidelines, which could complicate care pathways for patients.

The lack of an overarching prescribing strategy was partly attributed to differences in prison estate capacity, security category and nature of the confined population. Whilst local practices were sometimes needed to address local population needs, some participants perceived that this way of working restricted implementing standardised ways of working, such as safety interventions or individualised risk assessments protocols. Without overarching strategies, variable prescribing practices across prison sites ensued, and participants attributed this to personal preference, local culture, lack of knowledge or time pressures.

“*Since I have become a locum the thing that I hadn’t realised is the huge inconsistency from site to site …*. *you’ve got the same people working site to site under different rules on different days of the week*. *It is incredibly confusing*. *So you have similar patients*, *similar situations*, *different rules*, *not sure why*. *Very simple example*, *I’ve just come from somewhere that won’t give paracetamol in possession at all*, *but will dish out 84 ibuprofen*.” *SSI R 13*, *General Practitioner*

Whilst variations in practice are not unique to prison practice, when coupled with other challenges such as increased needs of patients that are prisoners, transient populations, staff retention, poor skill mix, problematic IT interface or tradability (discussed later), the potential of these variations to impact on prescribing is greater.

#### Prison structure

All participants described how the prison type, changeable prison population and prison regime contributed to prescribing challenges.

In addition to capacity, security category and population type (remand vs sentenced, longer stay vs short stay, male vs. female), meant healthcare teams were presented with transient patients and dynamic prison populations. Prison regimes and staffing capacity were reported to influence access to healthcare in several ways: restricted medicines administration times, reduced prison officer capacity to manage/supervise medicines administration queues.

“*they don’t have an across the board policy*. *So* …*We have 39 prisons doing 39 different things……because every regime in a prison is different*. *Every layout is different*, *every head of healthcare*…*for example*, *I don’t have a lot of sex offenders*, *but if you have sex offenders they have to come down at a different time to other prisoners*. *So it’s very much driven by a lot variables within the prison*.” *SSI R1*, *General Practitioner*

Occasionally, this was related to various organisational or operational barriers: shift changes for prison officers, prisoner working hours, visits from family/social workers/lawyers, cell searches and incidents of lockdown or segregation. As a consequence, GPs in particular reported high rates of patients not attending appointments across prisons, for reasons that would not present in general practice.

“*there’s a lot of gangsters in the prison*. *They have self-isolated because they are worried that if they come out of the wing somebody will attack them*. *Sometimes they are in the segregation unit from the night before and we don’t know until the next day*.” *SSI R11 General practitioner*

#### Staff retention, training and skill mix

Many participants cited the lack of robust training as a contributor to prescribing challenges. This included a lack of training for GPs starting at the prison and resulted in incidents being described such as off-label/inappropriate prescribing and poor documentation. A challenge unique to prisons was prison officers’ inability to discern between ‘drugs’ suspected to be of abuse and medication, leading to, patients not having access to analgesia or insulin.

Participants identified staff retention as a challenge caused by a lack of funding and the enhanced security clearance required to work in prisons.

“*Retention is difficult*. *It’s difficult to find staff that have got clearance*. *We’re category A so we need staff with counter-terrorist clearance and level two prison clearance*, *which is like a higher category of clearance*.” *SSI R3*, *Pharmacist prescriber*

Shortages, in particular for experienced GPs, nurses and clinically-focussed (rather than dispensing supply) pharmacists were perceived to increase workload pressure, impair patient centred care and the quality of prescribing. In many prisons, GPs in particular typically worked as locums, with little to no overlap in working hours for handover, which participants felt contributed to prescriber apathy, a lack of prescriber continuity, inappropriate prescribing, lack of adequate follow up/monitoring and medication safety incidents. While this may not be unique to the prison setting, the sense of silo-working compounded prescribing challenges for patients when prescribers attempted to rationalise other prescribers’ decisions. Participants also suggested that in the prison context, the sub-contracting of specialist services such as substance misuse, psychiatry or pain clinics, added to the complexity of prescribing and medication monitoring.

#### IT systems and interface

Despite using the same electronic health record (EHR) system (SystmOne^®^ (TPP)) across all prisons, participants identified challenges associated with this EHR’s use, and its poor interface with external systems. Participants reported variations in how sites recorded information on the EHR (for example, medication monitoring results were documented differently), which impaired the reliability and accuracy of prescribing.

“*Big problem with it is almost everyone enters everything free text*. *A lot of places will use [a] template*, *say here mental health use their template*, *substance misuse team*, *use their template*. *If they go to a prison that their organisation doesn’t cover*, *that new organisation won’t be able to access those templates*.” *SSI R10*, *General Practitioner*

*I know the [IT] system very well now*, *so maybe it’s easy for me*. *But the people who come*, *the locums*, *we have had a few locums and they struggle with the work*. *They struggle with the work because they don’t know*. *For them all the guidelines are new*, *prescribing information and non in possession*, *[knowing what is prescribed] weekly*, *daily*…. *very confusing for them*. *SSI R11 General practitioner*

The use of free text was attributed to a lack of familiarity with IT systems by locum or new staff, prescriber apathy or inability to access diagnostic information. A common challenge reported with using the EHR was the inconsistency with which read codes (a list of clinical terms for use by healthcare professionals to describe patient care and treatment) were used to document medical conditions and to link prescribed medication. This was reported to be more problematic in prison than general practice and could subsequently hinder identifying potentially hazardous prescribing or a lack of monitoring.

“*I mean*, *[if] someone’s on an antipsychotic in my community practice there would have been a diagnosis on the problem saying schizophrenia*, *so you could actually see what was wrong with the patient*. *The prison has no such information often about anything*…..” *SSI R10*, *General Practitioner*

Prescribers further highlighted that the lack of seamless interface between prison health and external records delayed effective verification of medication records at reception and clear communication at discharge and transfer of care.

#### Polypharmacy and comorbidity

All participants stated that polypharmacy and co-morbidity contributed to prescribing safety risks particularly among prisoners, because of the higher reported incidence of mental health/substance misuse problems in this demographic. Coupled in particular with the isolated nature in which some prescribers worked, clinical decision-making in prison settings brought added complexity.

“…*because of the complexity of our population behind the gate*, *I probably demand or expect a higher level of psychiatric oversight on medicines review for antidepressants*, *antipsychotics than perhaps I would demand or expect in a mainstream general practice*, *and that’s because I think our patients are more complicated*, *more subject to polypharmacy*, *may well have instances over their patient journey where they might have had a self-harm incident or a change in their presentation*….” *SSI R19 General practitioner*

Participants recounted how patients in the prison were often co-prescribed medicines affecting the central nervous system (CNS drugs), and raised concerns regarding the potential for adverse drug reactions, drug-drug interactions and increased morbidity/mortality risk through intentional or inadvertent overdose.

“*It’s challenging* …*a lot of patients is they’ve had injuries for years*, *five*, *ten*, *15 years* … *and because they’ve always been on some kind of pain relief*, *it’s very difficult to explain to them that being on it for such a long time is not actually going to have any benefits for your health*, *it’s actually making it a lot worse*. *And then you also have the substance misuse patients who have a history of substance misuse or are currently substance misuse patients and they are seeking more opiate based medication and you have to explain to them the risk of why we can’t supply it to them because we don’t want to put them at risk of harm and aid their addiction in a way*.…” *SSI R9 Pharmacist prescriber*

Attempts to mitigate these risks whilst addressing patient needs and adhering to prison-specific guidelines for example, safer prescribing in prisons of opioids for example added to prescribers’ perceived burden of responsibility. An increased sense of responsibility and liability was also attributed to providing healthcare in a system where resources to inform prescribing were often incomplete, conflicting or variable, patients were known to have higher mortality risk and coroner’s inquests were more prevalent.

#### Tradability

A significant concern voiced by all participants was the diversion of prescribed medication by patients for use as currency in the illicit drug trade. The tradability of a medicine influenced the risk assessment of its initial/continued prescribing both to prevent diversion of the medication, and to ensure safe, appropriate use.

“*As an example*, *zopiclone is highly tradable in the prison service*. *It feeds into the illicit economy; it goes for something like £5 or £6 a tablet on the wings*. *But genuine people with genuine insomnia obviously suffer*, *[*.…*] it makes no sense to give it to patients in possession*, *so you would always have it not in possession*, *which means they have to show up and collect their medication*. *And I must emphasise that the biggest driving factor behind all of this*, *which is absent in community or any other environment*, *is the illicit economy*. *It’s the tradability of prescription drugs … which wouldn’t necessarily be problematic in the community environment*. …. *there’s always a doubt in your mind*, *especially if you are aware of the individual and there could be a potential for abusing*.”

*SSI R2 Pharmacist non*-*prescriber*

In addition to diversion, tradable medicines promoted accumulation of prisoner debt and threats to personal safety, a problem seldom encountered in the general population. In seeking to mitigate behaviours fuelled by tradable medicines, a few participants acknowledged that sometimes patients suffered attacks following refusals to prescribe tradable medicines. Prescribers consequently felt a responsibility to consider safety of individual patients and the wider prison population when prescribing tradable medicines.

“*When you are prescribing as well you have to think about what you are prescribing*, *is it something that can be abused by the whole prison population or are you noticing a trend that more and more people are coming to ask you about this medication*.” *SSI R9*, *Pharmacist (prescriber)*

Despite this, most interview participants and all group discussion participants reported experiencing frustration when previously stopped or deprescribed tradable medications were later re-prescribed by external providers or community GPs.

“*Represcribing of pregabalin*, *tramadol et cetera by community GPs following release and detoxification in prison [is a common challenge]*” *NGD R4 Pharmacist (non-prescriber)*

*We had someone who we had started on a [gabapentinoid] reduction and he was near the end*, *he had only been out for a week and within that week was back on the maximum [dose]*…. *I think that’s the problem at the moment*, *there is no real communication between prisons and primary care and secondary care*. *The information is not being shared about what goes on in prison*. *SSI R9 Pharmacist prescriber*

#### Patient behaviour

Drug-seeking behaviour was most commonly referenced by all participants as a patient behaviour which challenged safe prescribing. Prescribers highlighted the need to understand how the novel misuse of prescribed and non-prescribed substances could occur in prison. Participants described experiences of violent, threatening and coercive behaviour from patients who were drug-seeking.

“*But they [diabetics] are the ones I am most uncomfortable with*. *I don’t particularly like an insulin-dependent diabetic holding me to ransom; but I’ve got about five or six that haven’t had their rivaroxaban for six months because they’re protesting*. *And they won’t take it until I get it [pregabalin] back to them*….” *SSI R1*, *General Practitioner*

Patients were also commonly reported to target prescribers perceived as easy to manipulate/intimidate, new-to-prison or willing to prescribe differently to their peers. Moreover, some patients targeted transfer of care periods to seek previously deprescribed drugs.

“*You’ll hear this almost unanimously from [prison] doctors who are prescribing*, *even if they’re deprescribing*, *it’s*, *‘Well don’t worry because when I get out my doctor will prescribe them for me’*. *So*, *it’s almost as if to say*, *‘Well I’ll suck it up for now*, *but you know*, *I’ll get my drugs back off the GP’*.*” SSI R20 General Practitioner*

Assessment of risky patient behaviour and the potential harms were discussed during the NGD and interviews; they included the risk of toxicity, self-harm and overdose following release from prison. Measures to mitigate these harms and reduce risky behaviour included prescribing in possession (IP) medicines, cell checks to verify medications quantities, drug screening, supervised consumption of medicines to monitor adherence, re-titrating or deprescribing tradable medicines.

#### Theme 3: Measures to address prescribing/medication monitoring challenges

All participants (in particular those working across different prison sites/estates) were in agreement that a ‘one size fits all’ approach to address prescribing challenges was unsuitable, due to the multifaceted, complex and variable nature of prison settings. Instead participants highlighted the need for solutions incorporating four distinct subthemes: collaborative working, consistency in practice, innovation, and education and training.

#### Collaborative working

Collaborative working protocols were most commonly reported by participants to improve communication and to reduce silo working.

“*two of our sites now have MDT prescribing meetings and they basically discuss people being caught diverting or they’ve had a lot around just looking at everybody who’s on pregabalin*, *gabapentin to try and unpick should they still be on it*. *But it’s multidisciplinary so the decision is being made jointly with the patient*, *so it gives security to the prescribers that they’re doing the right thing* …*SSI R14 Pharmacist non-prescriber*

The multi-disciplinary team (some of which included non-healthcare staff and external providers) approach was believed to support effective and objective clinical decision-making for complex patients; with initiating non-formulary prescribing, deprescribing tradable medicines and balancing recommendations from specialists (e.g. psychiatry, pain or substance misuse services) with the practicalities of prison prescribing (e.g. avoiding IP prescribing of flammables, ethanol diluents or injection devices). After the MDT came to a decision on an issue, patients were sent letters from the team and invited to face-to-face consultations. Here, an evidence-based clinical rationale would be used to explain the need for change to patients and the available options to manage their condition. Those prescribers described this approach as more patient centred, likely to mitigate challenges to prescribing decisions. They also believed that this offered an objective, acceptable consistent strategy to prescribing decisions.

“*We inform patients in writing*, *by letters*. *I’ve drafted plenty of letters in the past and sent them out to everyone that’s on that medication informing of them of a range of dates that it’s going to be happening and informing them that they have an opportunity to make an appointment to see the GP and discuss either alternative medication or any issues they’ve got* ….” *SSI R5*, *Pharmacist (prescriber)*

#### Consistency

The need for improved consistency was commonly discussed amongst all participants, especially where they felt inconsistency was detrimental to the provision of quality care: consistency in using read codes for clinical indications, consistency in staffing/service providers, continuity in practice across settings and at transfer of care, and consistency in documentation of test results/patient information.

“*Continuity of medicines on reception*, *release and transfer*, *and the consistent approach to medicines optimisation and medication review*, *in the main in association with is high quality long-term conditions management*.” *SSI R19 General practitioner*

There was consensus that blanket application of protocols/guidelines was inadvisable, because participants agreed it was important that guidance could be adapted for personalised care however a systematic approach between practitioners in applying prescribing options, protocols/formularies and thresholds, would help to address poor or potentially hazardous prescribing or monitoring practices which remained unchallenged. One doctor described developing a proforma, to standardise antipsychotic prescribing.

“*I devised it [the antipsychotic proforma] based on the NICE guidelines and so that’s what we used*. *And then we made the proforma*, *we showed it to the medical team and we showed it to the psychiatrists*, *they agreed with it*, *and now they use it*.” *SSI R11 General Practitioner*

#### Innovation

Innovative practice, incorporated improved skill mix of the MDT members and implementing new technologies. Expanding roles of clinical pharmacists in the prison setting was repeatedly identified as an innovative practice that enhanced prescribing and monitoring quality. This group of healthcare professionals were reported to conduct clinical audit, medicines review and optimisation, offer alternative treatment recommendations, repeat prescribing, and contributed to decision-making as part of the MDT.

“*I’ve been involved in a quality improvement project*, *looking at*, *again*, *as I mentioned*, *the tradability of psychotropics*, *hypnotics*, *sedatives*. *So*, *I’m thinking of introducing non-tradable alternatives*, *like melatonin*, *because it has zero tradability*, *they can keep it in possession*, *less operational pressure*.” *SSI R2 Pharmacist non-prescriber*

Further workforce and skill mix innovations included an increased pharmacy technician and paramedic workforce to support medicines reconciliation, out of hours urgent care and the timely administration of patient medications. Medicine lockers and dispensing machines located in communal areas were other strategies prisons used to widen access to healthcare.

One of the participants interviewed explained how the recent adaptation of the prison EHR by community GPs had minimised problems associated with information transfer for medicines reconciliation, discharge and continuity of prescribing/monitoring. Experienced clinicians viewed the EHR as an underutilised resource but with the potential to enhance clinical audits and identify potentially harmful medication usage.

Interestingly, the last two participants suggested that the onset of the COVID-19 pandemic, accelerated changes/innovations in practice such as a mass shift to prescribing IP and remote assessment. While this unprecedented change led to some initial anxiety about increases in overdoses, participants reported this increase did not occur and instead, various benefits were noted.

*What is interesting is*, *during COVID we’ve learned about* … *how that has impacted upon*, *not just the presentation levels of intoxication*, *but also the massive significant reduction in [risk] reviews and mental health presentation*, *self-harm*, *et cetera*. *So there’s definitely been a significant fall-off in substance misuse-related bullying*, *harassment and mental health presentations*, *it’s been dramatic*…*More so than I think anybody could have predicted*, *and it’s been sustained*, *so we’re now at 10*, *11*, *week 12*, *or coming up to week 12 [of the lockdown]*. *Now*, *I think there’s lots of thoughts and emerging reflections on that*.” *SSI R19 General Practitioner*

#### Education and training

There was widespread recognition amongst participants that improved training and incentivisation of prescribers could enhance the quality of prescribing. Unlike in primary care the Quality and Outcomes Framework (QoF) was not perceived to be a driver to improve prescribing quality. Prescribers therefore suggested linking prescribing directly to performance indicators, to incentivise their use.

“*we do use QOF within the prison setting and we’re seeing*, *although it doesn’t work the same way as it would do in community*, *in terms of there’s no financial incentive to us*, *but it does show a measure of how we’re managing long term conditions*, *so we are acknowledged that actually it is a helpful marker*” *SSI R12 Pharmacist non-prescriber*

Another measure promoted by some participants was to invest in the medicines use and safety training of prison officers, as those with greater involvement and awareness of health needs and medicines safety issues were perceived to better advocate for patients. Participants also felt that empowering patients to be involved in decisions about their care through education, collaboration and improved communication helped support challenging consultations.

“*I find that the reasons helps better*, *so I will often tell people*… *about the pregabalin*, *because it’s a big one…look*, *back when you were [first] given it the guidelines said give it to people*, *we didn’t know the dangers*. *You were a victim of our ignorance*. *We thought it was safer than it was and then people started dying*. *And now the rules have changed*, *so you’ve stood still*, *the world has changed round you*. *So it’s not your fault*. *But now the rules have changed round you because we know more*. *If you presented today like you did then you wouldn’t have got it*.” *SSI R13*, *General Practitioner*

## Discussion

This study explores the processes and challenges surrounding prescribing and medication monitoring in prisons, explore their causes and impact, and to identify potential approaches to improving prescribing safety. This study adds new knowledge to existing research by Bartlett *et al*., that has highlighted inconsistencies in prison prescribing, the increased mental health needs of prison populations and challenges including tradability [10]. Moreover, it reveals aspects of organisational safety culture in prisons, it contrasts elements of prison and community (general) practice to highlight problems unique to prison and provides insight into current innovations and measures (such as the inclusion of clinical and prescribing pharmacists) that could successfully be used in practice. Moreover, the proposed COM-B model offers a novel approach to developing tailored interventions to address prescribing challenges in prison. Some of the challenges reported in our findings are known to exist in general practice, however study findings highlight the nuanced differences of these challenges when the patient is a prisoner. More specifically, findings point out a number of prescribing challenges unique to the prison context. Patients who are prisoners are predisposed to socio-medical and psycho-social needs that require a holistic approach to prescribing and risk assessment. The contextual characteristics, workplace culture and variations in practice highlighted in this study are important to our understanding of the multifactorial nature of challenges to prescribing and monitoring in prison. It is also crucial to recognise that in addressing these challenges a ‘one size does not fit all,’ but with local understanding the suggestions we have identified may be adapted and taken forwards as part of remedial strategies. Findings from this study also advance the World Health Organisation’s Global Patient Safety agenda to characterise how the safety of prescribing may be compromised in prisons, and to inform suggestions for interventions for further testing.

Previous studies exploring ways to improve safety and quality in healthcare have emphasised the importance of understanding context [46, 47]. By definition, the prison refers to a state of forced confinement within which healthcare and prescribing must be delivered [37]. Our findings highlighted the peripheral positioning of healthcare in prison and the challenges presented in trying to deliver a prescribing model designed for general practice and the general population, that has elements of secondary or tertiary care incorporated into it. The Safer Prescribing in Prisons guidance describes this model as ‘equivalence’, which recognises that care provision in prisons requires a different service model that is not the same as care provided in the community [14]. With this in mind, it is important for healthcare staff to be trained to recognise that healthcare provision in prisons is unique and need to tailor the care provided to their patients without enforcing a model focused on primary, secondary or tertiary care.

In understanding the causes of and contributors to prescribing challenges in prison healthcare seven subthemes emerged, and were encompassed within the main themes of culture and variations in practice. There is research to suggest that culture and variations in practice (what people actually do in practice) are very closely linked; that is, the notion that culture plays an important role in how behaviours manifest [46, 48, 49]. A study by Kiran et al., 2019 proposes ten tips for advancing a culture of improvement, which includes clinicians and staff committing time and resources towards quality improvement work, appealing to the intrinsic motivation of staff to improve patient care, integrating quality improvement with management and operations, and measuring and improving patient experience [50]. Our findings support this by suggesting that operational changes, and modifications in clinician and staff skill mix and behaviours, can drive improvements in practice and organisational culture. One such example already implemented in some prisons, is using pharmacists to improve the quality of prescribing, a physical intervention that has been successfully used in general practice. Further examples are detailed in Table 1.

**Table 1:** Examples of reported interventions designed to improve prescribing safety/quality mapped onto COM-B.

**Capability**		
*Capability is physical or psychological*		
Improve the ability to make the change		
**Domain**	**Description of an intervention described in results**	**Challenge to safer prescribing or monitoring**
**Physical**	Clinical Pharmacists trained to directly order monitoring when needed so don’t rely on GPs or psychiatrists	Monitoring omissions for antipsychotics
	Prison officers trained to perform functional pain assessments	Lack of evidence (i.e. physical observation) for patients complaining of pain
	Change to use EHR that allows for interoperability/communication functions by all primary care organisations	Lack of IT interface for information transfer across from primary care
**Psychological**	Establish weekly MDT team for shared decision making for complex/challenging cases	Making challenging prescribing decisions in isolation
	Review of health literacy and patient education to support letters signed by all MDT issued to inform patient prior to face-to-face meeting and printed evidence-based rationale to discuss with patient	Concern regarding consequences of informing patient that pregabalin is being stopped
	Default use of ‘in possession’ medication for all patients	Patients inability to manage substance misuse medications on discharge, making this a high risk period
**Opportunity**		
*Opportunity is physical or social*		
Reduce triggers/opportunities to continue old behaviours and increase opportunities for change		
**Domain**	**Description of an intervention described in results**	**Challenge to safer prescribing or monitoring**
**Physical**	Improved skill mix by employing increased clinical pharmacists for medicines optimisation and pharmacist prescribers	Lack of qualified staff to run clinical audits to monitor quality of prescribing
	Skill mix improvement to better delegate work	High workloads and lack of staff to delegate to
	Medicine dispensing machines within prison wings	Lack of capacity at medicine administration time, inability to prescribe frequencies outside of prison regimes
	Use of compatible IT systems which are interoperable and can allow for efficient and timely communication by and between GPs in community and in prison settings	More seamless medicines reconciliation and discharge process
	Consistent staffing	Reliance on locum staff causing lack of follow up
	Training for prison officers on medicines use	Prison officers viewing medicines in a similar way to illicit drugs’
**Social**	Multi-disciplinary collaborative working and peer support meetings	Inter/intraprofessional conflict between internal and external providers, professional isolation
	IT analysts and clinicians collaborating to perform audits that will assist in identifying potential harms using the EHRs	Inability to efficiently perform audits to identify potential harms
**Motivation**		
*Motivation is reflective or automatic*		
Increase the desire to make the change now/soon		
**Domain**	**Description of an intervention described in results**	**Challenge to safer prescribing or monitoring**
**Reflective** Intentional or by choice	Training (and educating prisoners about medication use and self-care) and incentivization of prisoners (discourage aggressive drug-seeking behaviours with verbal reminders of ramifications or consequences)	Lack of awareness/unwillingness amongst patients towards deprescribing Prisoner perception of deprescribing as punitive
	Prioritise patient centred and health care more generally in the prison context	Acceptance of views that prison healthcare is not always of sufficient quality
	Structured training for new-to-sector prescribers around clinical decision-making	Variability in training or induction of doctors prescribing and managing medication in prisons, and challenges associated with it Doctors trained/inducted to new settings by healthcare assistants
**Automatic** Habit or impulse	Improving how clinicians utilise IT systems–introducing standard operating procedures in the use and documentation of information	Inability to correctly document or locate information or order tests on the systems
	Linking of performance indicators and prescribing improvement initiatives	Lack of incentivization for quality improvement measures
	Use of evidence-based quality indicators to rationalise prescribing and rationalise decisions in coroner’s court	Weight of responsibility in justifying decision-making during coroner’s court

**Fig 2:**
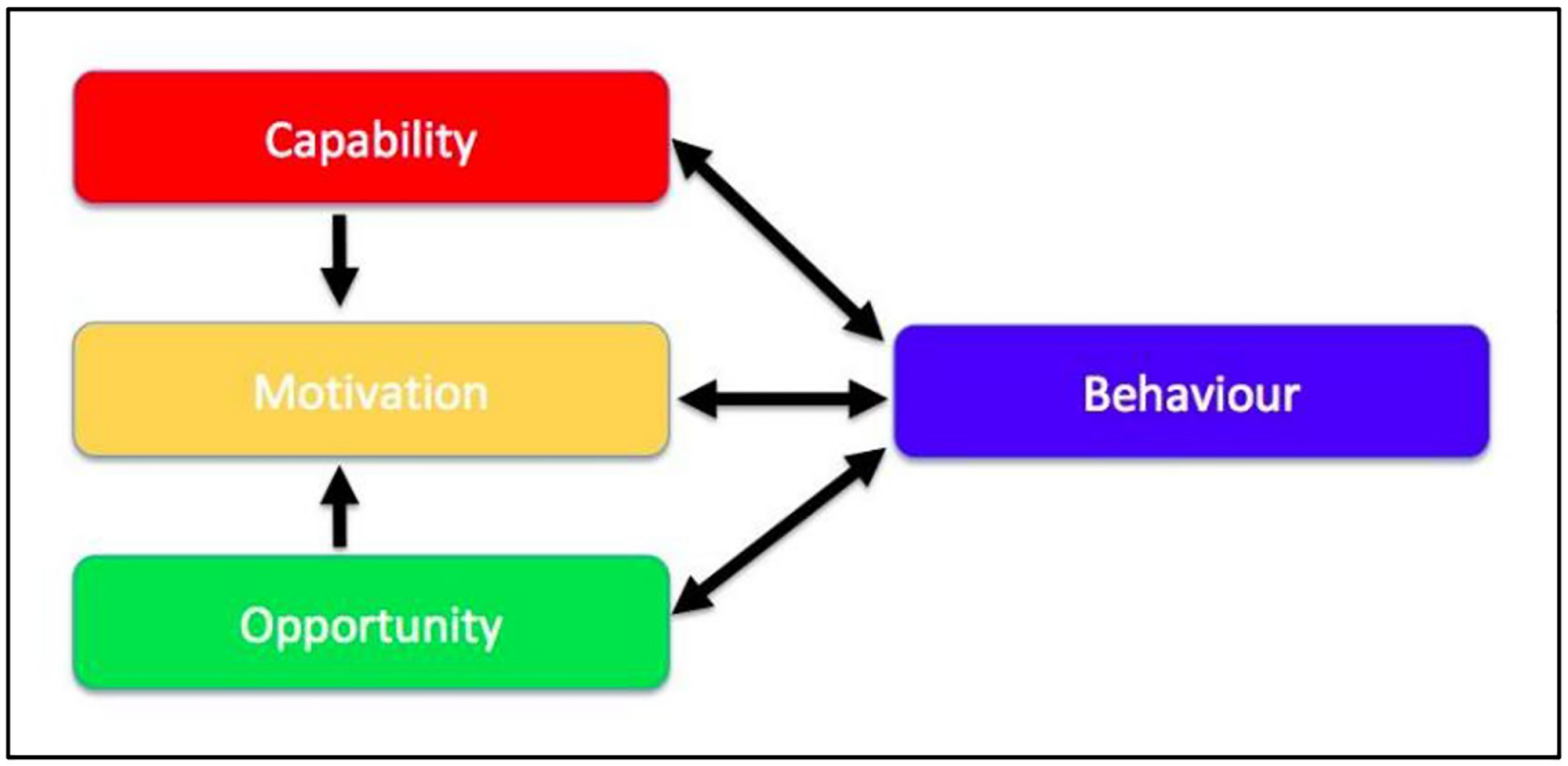
COM-B model (Michie et al.) [43].

Participants provided examples of approaches to improve prescribing quality/safety that some had first-hand experience of, and which focused on changing ways of working (collaborative working, communication and consistency). Studies have shown that multidisciplinary collaborative working could improve prescribing quality/safety [22, 51, 52]. In a cluster randomised trial known as ‘PINCER’, results showed that a pharmacist-led intervention in collaboration with doctors reduced a range of medication errors in general practice [22]. Central to participant accounts was a necessary shift in workplace culture and prescribing behaviour to support their success, and a recognition for and willingness to use education and training to change ways of working. The willingness and readiness to change of an organisation has long been regarded as crucial to achieving culture change in patient safety. Findings from our study highlighting what may need to change may therefore be used by organisations ready to move forwards to develop a behaviour change intervention that is effective. To understand the implications of our findings, the Capability, Opportunity, Motivation–Behaviour model (COM-B), a widely-used theory of behaviour change can be applied [53, 54]. (See Fig 2)

Developed by Michie et al., COM-B proposes that for an intervention to be successful, people need capability, opportunity and motivation to perform a desirable behaviour [53, 55]. A review of the main findings is useful to discern areas where prison healthcare staff lack capability to improve the safety of prescribing, how they can identify potential opportunities to improve the safety of prescribing and increase motivation for safer prescribing. Performing the desirable behaviour is possible when physical and psychological capability, social and physical opportunity is present and when both reflective and automatic motivation are facilitated. Where undesirable behaviour is identified, understanding and analysis of the determinants of that behaviour are useful to help identify what then needs to change. In this way, the model is useful for understanding of behaviours in context and then developing a new targeted behaviour, as a basis for intervention design.

When applied to our findings, COM-B provides some understanding of how a specific improvement intervention described by participants may lead to behaviour change. For example, participants that reported having regular MDT meetings had the physical capability and opportunity to hold meetings which empowered them (psychological capability) and motivated them to provide improved care. This is further illustrated in Table 1, which assigns interventions raised by participants to specific prescribing and monitoring challenges and a COM-B domain. Organisations with an understanding of their own unique challenges may similarly use this framework to identify and review an existing prescribing or monitoring challenge, assign it to an area of COM-B and develop an intervention based on this.

To our knowledge, this is the first study reporting detailed perceptions from multidisciplinary clinicians of how prison settings, prison regimes/systems, and prisoner/prescriber behaviours influence safe prescribing, as well as the measures used to mitigate this.

The strengths of this study include the recruitment of a wide range of professional groups at a national level from prison settings. The expert panel and semi-structured interview participants involved specialised healthcare professionals from varied backgrounds with considerable experience providing care for remand, convicted, male, female and Category A-D prisoners, which provided different views on prescribing challenges within prisons. This allowed us to reach data saturation from our analysis.

One of the limitations of our qualitative approach was the lack of participants from some settings, which meant that views particular to female prisons or more specialist secure settings (such as youth offender settings) may have been missed. For example, while four participants reported having experience of working in women’s prisons, none were currently working there. In addition, participants who took part in our study were likely to have an interest in medication safety and may not represent the views of all healthcare professionals working in prison settings. One participant participated in both the NGD and interviews, which may have introduced bias. To reduce bias this participant was interviewed after at least 10 other participants and themes from their interview analysis were observed to be consistent with those of other participants. There may also be elements of self-selection, social desirability and recall bias which may have led to an over-reporting of ‘good’ behaviours and under-reporting of poor practice [56]. This could be mitigated with modified wording in the interview guide, a reminder of participant confidentiality and ensuring participants know that the interviewer was a pharmacist, they had no experience of work in the prison setting. A lack of time prevented transcript checking and verification of findings from interview participants as well as repeat interviews, which may have supported triangulation.

## Conclusion

When making efforts to improve medication and prescribing safety in prison, organisations should gain a clear understanding of the process and challenges associated with prescribing in their local prisons. This should include consideration of the unique needs of the patient as a prisoner, as well as the variation and culture of the prison context. Our findings indicate that ‘a one size fits all’ approach will not work; instead the COM-B framework may be applied to successfully develop a bespoke intervention that is suitable for local implementation.

Future research should explore the views of prisoners who are patients, and prison officers, external healthcare providers and community prescribers about medication and prescribing safety. This may inform improvements to healthcare access in prison and at transitions to and from prison. Furthermore, research is needed, to explore how information technology could be better utilised in improving the healthcare of patients who are prisoners.

## Supporting information

S1 File Nominal group question and prompts. (DOCX)

S2 File Interview guide V5. (DOCX)

S3 File Prison categories. (DOCX)

S4 File (ZIP)
