# Supplementary material for: Exploring the challenges to safer prescribing and medication monitoring in prisons: A qualitative study with health care staff
Source: PLoS One. 2022 Nov 3;17(11):e0275907. doi: 10.1371/journal.pone.0275907 (PMC9632766; doi:10.1371/journal.pone.0275907)
Supplement: S1 File — (DOCX) [file pone.0275907.s001.docx]

**NOMINAL GROUP QUESTION AND PROMPTS**

***"What medication-related errors/harms or examples of hazardous prescribing are most likely to occur in the prison setting and what is their potential severity?”***

**Prompts**

Is there anything else you would like to say about that?

What do you mean when you say that?

Can you give us a few examples to further explain wat you mean?

What did you do/say/think when that [incident/event] happened?

Have any others had the same experience?

Have any others had a different experience?
