# Supplementary material for: Exploring the challenges to safer prescribing and medication monitoring in prisons: A qualitative study with health care staff
Source: PLoS One. 2022 Nov 3;17(11):e0275907. doi: 10.1371/journal.pone.0275907 (PMC9632766; doi:10.1371/journal.pone.0275907)
Supplement: S2 File — (DOCX) [file pone.0275907.s002.docx]

# Appendix 3 Semi-structured interview schedule

**A study to explore the feasibility of prescribing safety indicators (indicators of potentially hazardous prescribing and medication monitoring practice) for prison settings**

**The interviewer will introduce themselves and the purpose of the interview using the script below.**

**Interviewer introduction:**

I am Dr Esnath Magola a researcher at The University of Manchester. I am part of a team carrying out a project which has an overall aim of developing indicators which effectively identify patients who may be exposed to potentially hazardous prescribing and drug monitoring, and to facilitate improvement. These indicators have been called prescribing safety indicators. We have developed a potential suite of PSIs for testing in the prison setting and this study will contribute to testing the feasibility of prescribing safety indicators (PSIs) in prisons.

However, before we do that it is important to explore how medication safety is perceived and managed in prisons as there is a lack of published research on this subject. This interview aims to explore your views about challenges to safer prescribing, what you think are the causes/contributors to prescribing and drug monitoring safety challenges, and potential solutions to address those challenges. We would also like to explore how prison health care staff might use PSI data in the workplace and what factors may influence this. The interview will last up to one hour. Findings from this interview will be used to inform recommendations for implementing and responding to PSIs in prison settings.

I should remind you that the interview is confidential and that you refrain as much as possible from naming people or places. I’d like to audio record the interview if that is okay with you; this is simply to help me accurately capture all of the information that comes out of it. You can ask for the audio-recorder to be switched off at any point during the interview. The audio recordings will be destroyed once transcribed and checked by the research team. Interview transcripts will be kept in a secure University location for five years after the findings of the study are published before being destroyed.

**SCHEDULE (total up to one hour)**

**Pre-interview**

An email containing some reading material was sent to you earlier to help you understand the purpose and use of patient safety indicators for safer prescribing. Do you have any questions about any of this material? We can spend 5 minutes clarifying your understanding of prescribing safety indicators before we begin the interview.

**Part 1 Interview: Introduction**

- Could you tell me a little bit about your current role in the prison setting and how long you have worked there?
- What involvement do you have with medication management, medication safety and/or prescribing and drug monitoring?

**Part 2 Challenges to medication/prescribing safety**

[Prescribers]

- Could you describe the physical processes you go through when prescribing a medication for a prisoner? (including environment, safety level of surroundings, physical barriers to prescribing)
- Could you describe the cognitive (thought) processes you go through when prescribing a medication for a prisoner? (including motivations, views, values, beliefs, use of guidelines, compare ‘new item’ vs ‘repeat item’, in-possession vs. not in-possession medication)
- What do you feel influences your prescribing/monitoring decisions? Prescriber desire/need? Best for patient/organisation/guidelines/practice? Fear of negative consequences? Culture/habit/workload pressures or demands? Level of training/understanding an SOP? Patient behaviours or pressure?
- How do you respond to/ manage on screen alerts and what influences this?
- How do you use medication monitoring data on prisoner records?
- How well do you know the prisoners you are prescribing for? Does that influence you prescribing decisions?
- Currently, what systems do you have in place to identify patients who are at risk of harm?

[Prescribers and Non-prescribers]

- In your view, what are the most important prescribing and drug monitoring safety challenges in the prison setting?
- What is causing these medication safety challenges in prison?
- What is contributing to/influencing those challenges? [Break this down into those challenges relating to processes, systems, behaviours/communication of practitioners/prisoners]
- What is the impact of these challenges on prisoners, staff, organisation?
- What (potential) harms do the medication safety challenges lead to and
- How do you currently identify and monitor these harms?

**Part 3 PSIs and medication safety**

Have a look at the PSI examples we sent to you, to help you understand the purpose and use of patient safety indicators for safer prescribing, which is to help identify patients who are at risk of harm. We would like you to think about those statements, and using them in practice.

- For the following PSIs:
- Would you want to access PSI data like this? How would you want to access it?
- How would you go about reviewing it/responding to the data?
- What kind of impact do you think this would have – on staff, on prescribing, on workload on patient safety?
- What would prevent you from using PSIs like this in your prison?
- What would help/support you to use PSI like this in your prison?

**Part 4 Solutions to prescribing and drug monitoring safety challenges**

- How do you address these challenges when they arise in prison? What is the process?
- What more should/could be done to ***prevent*** these prescribing/monitoring safety challenges in prison?
- What more should/could be done to ***address*** prescribing/drug monitoring safety challenges in prison?

**Interviewer conclusion (5 minutes)**

Concluding questions:

- Is there anything else that you would like to add?

Switch off tape recorder. Many thanks for taking the time to help us with the Medication Safety in prisons study. Your contribution has been extremely valuable. We will contact you in future to ask whether you would like to receive a summary of the findings of this research study. In the meantime, please feel free to contact either myself or another member of the research team if you have questions or other issues to discuss.
