# Supplementary material for: Exploring the challenges to safer prescribing and medication monitoring in prisons: A qualitative study with health care staff
Source: PLoS One. 2022 Nov 3;17(11):e0275907. doi: 10.1371/journal.pone.0275907 (PMC9632766; doi:10.1371/journal.pone.0275907)
Supplement: S3 File — (DOCX) [file pone.0275907.s003.docx]

**Prison categories: supplementary file**

Source of information: <https://prisonjobs.blog.gov.uk/your-a-d-guide-on-prison-categories/>

Prisons in England and Wales are categorised in different security categories ranging from category A (highest security) down to category D (lowest security).This categorisation is based on:

- risk of escape
- harm to the public, if they were to escape
- threat to the control and stability of a prison

**Category A: high security**, housing male prisoners who, if they were to escape, pose the most threat to the public, the police or national security.
**Category B: local or training prisons**, housing prisoners that are taken directly from court in the local area (sentenced or on remand), and training prisons hold long-term and high-security prisoners.
**Category C**: **training and resettlement prisons**, housing prisoners with the opportunity to develop their own skills so they can find work and resettle back into the community on release.
**Category D: open prisons,** with minimal security and allowing eligible prisoners to spend most of their day away from the prison on licence to carry out work, education or for other resettlement purposes.
