# Supplementary material for: Exploring the challenges to safer prescribing and medication monitoring in prisons: A qualitative study with health care staff
Source: PLoS One. 2022 Nov 3;17(11):e0275907. doi: 10.1371/journal.pone.0275907 (PMC9632766; doi:10.1371/journal.pone.0275907)
Supplement: S4 File — (ZIP) [file pone.0275907.s004.zip › Supplementary file - Harms/Supp file 7.1 Paper 1 Pre-reading Spencer_.pdf]

Rachel Spencer, Brian Bell, Anthony J Avery, Gill Gookey and Stephen M Campbell

Identification of an updated set of prescribing-safety indicators for GPs

Abstract

Background
Medication error is an important contributor to patient morbidity and mortality and is associated with inadequate patient safety measures. However, prescribing-safety tools specifically designed for use in general practice are lacking.

Aim
To identify and update a set of prescribing-safety indicators for assessing the safety of prescribing in general practice, and to estimate the risk of harm to patients associated with each indicator.

Design and setting
RAND/UCLA consensus development of indicators in UK general practice.

Method
Prescribing indicators were identified from a systematic review and previous consensus exercise. The RAND Appropriateness Method was used to further identify and develop the indicators with an electronic-Delphi method used to rate the risk associated with them. Twelve GPs from all the countries of the UK participated in the RAND exercise, with 11 GPs rating risk using the electronic-Delphi approach.

Results
Fifty-six prescribing-safety indicators were considered appropriate for inclusion (overall panel median rating of 7–9, with agreement). These indicators cover hazardous prescribing across a range of therapeutic indications, hazardous drug–drug combinations and inadequate laboratory test monitoring. Twenty-three (41%) of these indicators were considered high risk or extreme risk by 80% or more of the participants.

Conclusion
This study identified a set of 56 indicators that were considered, by a panel of GPs, to be appropriate for assessing the safety of GP prescribing. Twenty-three of these indicators were considered to be associated with high or extreme risk to patients and should be the focus of efforts to improve patient safety.

Keywords
ambulatory care; consensus; general practice; medication errors; patient safety; primary health care; quality indicators.

INTRODUCTION

Prescribing medications is one of the most powerful tools available to GPs in the prevention and treatment of disease, and alleviation of symptoms. Nearly 961.5 million community prescriptions are dispensed annually in England.[1] However, medication-related adverse events arising as a result of primary care prescribing are an important source of patient morbidity, much of which could be prevented by the highest quality prescribing and medicines management.[2] Prescribing-safety indicators are statements describing prescribing events that put the patient at risk of harm. Using these statements to audit or analyse clinical records is a powerful way for GPs to conduct focused and high yield-for-work audit activity on their own prescribing.

Worldwide, there have been many attempts over the past decade to develop prescribing indicators (mostly using consensus methods) for use in non-specific settings.[3–11] Most of the indicator sets developed specifically for primary care originated in the UK.[12–17] Other methods of analysing prescribing also exist, for example, prescribing analysis and cost tabulation (PACT) data and the Medication Appropriateness Index (MAI).[18,19] There are obvious limitations with PACT data because of a lack of linkable clinical information, and the MAI is a time-consuming process involving detailed medical records analysis. With the development of methods for interrogating electronic medical records,[20] there is now the opportunity in the UK to develop and use sophisticated indicators that can give a rapid assessment of the quality and safety of prescribing in individual practices using 'plug and play' software similar to that used in Quality and Outcomes Framework (QOF) assessment. This study aimed to update and expand, to whole-practice applicability, a set of prescribing-safety indicators (previously developed by some of the authors in 2009[15]) for assessing the safety of prescribing of individual GPs in UK general practice. It also aimed to identify which of these indicators were associated with the greatest risk of harm to patients.

METHOD

Identification of indicators

An extensive literature review of tools for patient safety in general practice was used to source indicators. Two independent reviewers followed Cochrane guidelines for systematic reviews[21] (for a full description of the search strategy see Appendix 1). Search terms in three stems (setting, topic, and output) were performed on the following databases: Embase, CINAHL, Pubmed, MEDLINE (Ovid 1996 onward), Health Management Information Consortium, and Web of Science on 1 November 2011. The

R Spencer, BMBS, BMedSci, academic clinical fellow; **B Bell,** PhD, postdoctoral researcher; **AJ Avery**, DM, professor of general practice, Division of Primary Care, School of Medicine, University of Nottingham Medical School, Queen's Medical Centre, Nottingham. **G Gookey**, BPharm, primary care pharmacist, NHS Rushcliffe CCG, Ruddington, Nottingham. **SM Campbell**, PhD, professor of primary care research Centre for Primary Care, Institute of Population Health, University of Manchester.

Address for correspondence

Brian Bell, Division of Primary Care, School of Medicine, University of Nottingham Medical School, Queen's Medical Centre, Nottingham, NG7 2UH, UK.

E-mail: brian.bell@nottingham.ac.uk

Submitted: 27 September 2013; **Editor's response:** 28 October 2013; **final acceptance:** 2 January 2014.

©British Journal of General Practice

This is the full-length article (published online 31 Mar 2014) of an abridged version published in print. Cite this article as: **Br J Gen Pract 2014; DOI:10.3399/bjgp14X677806.**

How this fits in

GPs have a very important role in improving patient safety by carefully prescribing and monitoring patients' medicines. Nevertheless, many patients are put at risk, and some are harmed, as a result of hazardous prescribing in general practice. Assessing the safety of prescribing by GPs is an important feature of patient safety measures. This study updates the prescribing-safety indicators developed by the authors in 2009 for the Royal College of General Practitioners, and identifies which of these indicators are most likely to lead to harm to patients.

output was too heterogeneous to apply meta-analysis techniques, so the study considered individual indicators using the following process. First, potential indicators that described a pattern of prescribing that could be hazardous and may put patients at risk of harm were identified and then the exclusion criteria shown in Box 1 were applied. New indicators found in the systematic review process were added to an existing set of 34 indicators published by the authors.[15] The literature was re-reviewed particularly to identify new indicators published since 2009.

The indicators developed in this project were derived mostly from existing sources because of the considerable amount of work that has been done on prescribing-safety indicators in other countries and other settings. In addition to the sources for the Royal College of General Practitioners (RCGP) indicators,[3–5] a number of new key papers were found that were previously unknown to the authors (Table 1).[6–11,17] Over 600 prescribing indicators were reviewed against the inclusion and exclusion criteria, and against attributes of good indicators, including importance, validity, and feasibility of data collection using electronic health records. After removing duplicates, 34 original RCGP indicators and 37 new indicators were considered suitable for inclusion in round 1 of the subsequent consensus technique.

There are various reasons why few candidate indicators were chosen from some of the sources. For example, many indicators from the Screening Tool of Older Person's Prescriptions[5] relate more to the appropriateness of prescribing than to safety. The Beers criteria[3] relate more to US prescribing during the 1990s than they do to the current UK situation. Reasons for not selecting more indicators from new sources are listed in Table 1. Grey literature from patient safety organisations, such as the National Patient Safety Agency and the Agency for Healthcare Research and Quality, and targets from sources, such as the QOF and the National Service Framework for Older People, were also examined for potential indicators.

Defining the indicators and evidence base

Electronic searches of the literature were conducted and respected reference sources, such as the *British National Formulary*,[22] *Stockley's Drug Interactions*,[23] and *Martindale*[24] were drawn on. The supporting evidence base was summarised by a clinical pharmacist as a synopsis for each of the potential indicators and was also used to inform the rewording of indicators, or in some cases, generate new indicators. In most cases, indicators were defined according to the wording used in previously published studies and reports but, in some instances, this was altered either to make the indicators more relevant to UK general practice or to give more specific detail regarding the drugs and conditions covered. For eight of the new indicators, one or more variations in the text were produced; this resulted in a total of 48 indicator statements for the 37 indicators. As an example (see next page), the original indicators (a) and (b) (drawn from Guthrie *et al*'s[17] and Zhan *et al*'s[10] indicators) were presented to the panel as the two original statements and also in a format suggested by the research team (c). The panel eventually refined the indicator to version (d), which achieved consensus.

Box 1. Inclusion and exclusion criteria

Inclusion criterion

1. The indicator describes a pattern of prescribing that is potentially hazardous and may put patients at risk of harm.

Exclusion criteria

1. The indicator describes a pattern of prescribing that is so unusual in UK general practice that the yield is likely to be too low to justify inclusion in the indicators set.
2. Extraction of data required for the indicator (from general practice electronic health records) is unlikely to be feasible.

Table 1. Source descriptors of indicators

Source (by age of publication, most recent first)	Indicators reviewed from source	Indicators used in round 1 of the consensus process	Source details	Relevance to prescribing safety indicators
PINCER trial:[16] cluster randomised pharmacist intervention trial	11	6	Outcome measures of the trial used as indicators	Used to inform the 2009 RCGP indicators
Guthrie *et al*:[17] Scottish general practice prevalence of inappropriate prescribing to vulnerable patients	15	12 (some crossover with other sources)	New paper published since the RCGP indicator set was developed, indicators taken from consensus	All were considered related to safety but some overlapped with the 2009 RCGP indicators
Wessel *et al*:[6] prevalence of prescribing and monitoring errors	30	11 (some crossover with other sources)	Data from US GP patients, indicators developed by the research team	Some drug–disease combinations represented quality rather than safety
NORGEP:[7] potentially inappropriate prescriptions for older people	36	5 (all of these were also found in other sources)	Based on Norwegian consensus panel	Many drugs are not relevant to the UK or are no longer prescribed
STOPP/START:[5] Irish prescribing indicators	65	27	Many relate to appropriateness rather than safety	Used to inform the 2009 RCGP indicators
Basger *et al*:[8] Prescribing Indicators Tool for Elderly Australians	48	4 (all of these were also found in other sources)	Based on the most commonly prescribed drugs to Australians aged >65 years	Many indicators reflect quality, especially secondary prevention of cardiovascular disease
Raebel *et al*:[9] laboratory safety monitoring in ambulatory patients	9	3 (some crossover with other sources)	Computerised tool used on US GP records	Some recommendations were considered to have too little evidence in their favour
Zhan *et al*:[10] Potentially harmful drug–drug and drug–disease combinations	56	No formal indicators but 3 co-prescribing statements related to warfarin	Data from elderly US outpatients, indicators from consensus	Many drug–disease combinations represented quality rather than safety
PDRM:[14] Manchester indicators on preventable drug-related morbidity	29	4	Successfully tested in English GP surgeries	Used to inform the 2009 RCGP indicators
Beers 2003 update:[3] US indicators of prescribing safety in older people	89	20	Although updated again in 2012 these indicators are US-specific and many cover situations not felt to be of high clinical importance	Used to inform the 2009 RCGP indicators
ACOVE:[4] Assessing Care of Vulnerable Elders	217	2	Many process measures, minority related to safety	Used to inform the 2009 RCGP indicators
McLeod *et al*:[11] defining inappropriate prescribing practices for older people	71	6 (all of these were also found in other sources)	Canadian consensus process dating from 1997	Many indicators were outdated (new pharmacological evidence)

a. Tricyclic prescribed to a patient with heart failure.

b. Tricyclic prescribed to a patient with an arrhythmia, heart block or postural hypotension.

c. Amitriptyline at dose >75 mg prescribed to a patient with the above cardiac problems.

d. Amitriptyline at dose >75 mg prescribed to a patient with heart failure, arrhythmia, heart block or postural hypotension.

Consensus process

The RAND Appropriateness Method, which is an established approach for the development of health indicators,[25–28] was used to select the most appropriate indicators. This method seeks to combine scientific evidence with the collective judgement of experts: a consensus opinion is derived from a group, with individual opinions aggregated. An attempt was made to re-recruit the 12 panel members who met and agreed on the 2009 RCGP indicators[15] to achieve consistency in reconsidering previously published indicators for a new purpose. Nine members of the original panel participated and were joined by three local GPs recruited from the Vale of Trent Faculty of the RCGP. The following factors were taken into account when selecting panellists: professional background (practising GPs), employment status (a range of different types of GP, such as, partners, salaried, and so on), sex, geographic location (GPs represented each of the four countries of the UK), and professional roles (some GPs with experience of working as appraisers and/or assessors of GPs). It was estimated that each member of the panel committed at least 3 days of work to the consensus-building exercise; they were partially reimbursed for their time. A further exercise was also conducted to assess the risk to patients associated with the indicators that were selected by the RAND panel (two additional GPs with expertise in prescribing who were used to replace two panellists who unfortunately could not participate in this later exercise).

Data analysis

This study adhered to the RAND Appropriateness Method[25] by conducting a two-round consensus process. In round 1, which was conducted by email in July 2012, panel members were asked to consider each indicator on its own merits using the summarised evidence for each as well as their own experience as practitioners. Panellists were asked to consider separately the suitability of each indicator for assessing the safety of individual prescribers and for assessing the safety of the practice as a whole. In round 2, panellists met for a 1-day face-to-face meeting in July 2012, where under the chairmanship of two moderators (one with extensive experience in RAND panel methodology), they discussed each indicator in turn as a group and then re-rated the indicators on individual rating sheets. These round-2 rating sheets included the panellist's own rating on round 1, and for comparison, presented the frequency distribution of ratings of all panellists (anonymised) and the overall panel median rating. During round 2, panellists also had the option to propose alternative wording for indicators, which they would then refine by consensus decision. In both rounds, panellists were asked to rate each indicator on a nine-point scale. A rating of 1 meant that it would be extremely inappropriate to use the indicator, whereas a rating of 9 meant its use would be extremely appropriate. The overall panel median ratings were as follows: 1–3: inappropriate; 4–6: equivocal, or unsure of appropriateness; and 7–9: appropriate. The level of consensus within the panel for each scale for each indicator was also calculated. Agreement signified that no more than 20% of panellists' ratings were outside the same 3-point region (that is, 1–3, 4–6, 7–9) as the observed median (that is, for a 12-person panel no more than two ratings outside this 3-point region). This method was identical to the one used in the authors' previously published research.[15] Results are presented for the final (round 2) ratings only.

A subsequent Delphi methodology exercise asked the panellists to rate the potential harm to patients and the likelihood of hazardous prescribing for those indicators that had been considered appropriate by the RAND panel. A single round was conducted by email. Harm was rated on a 5-point scale that ranged from 1 (insignificant) to 5 (catastrophic); likelihood was rated on a 5-point scale that ranged from 1 (rare) to 5 (almost certain). Panellists were asked to use the information provided in a background document and their own experience as clinicians to rate the indicators. They were also provided with information on how commonly the drugs were prescribed in England in 2011 (prescriptions dispensed per 1000 patients). Panellists were invited to provide comments, but were asked to rate the indicators as they were written. Harm and likelihood scores from each panellist were multiplied to put each indicator into one of four risk categories.[29] The risk categories were: 1–3 (low risk), 4–6 (moderate risk), 8–12 (high risk), and 15–25 (extreme risk). Indicators were considered high or extreme risk when the median risk category for that item was high or extreme and 80% or more of the participant scores were in the same risk category as the median.

RESULTS

Figure 1 shows the steps taken in arriving

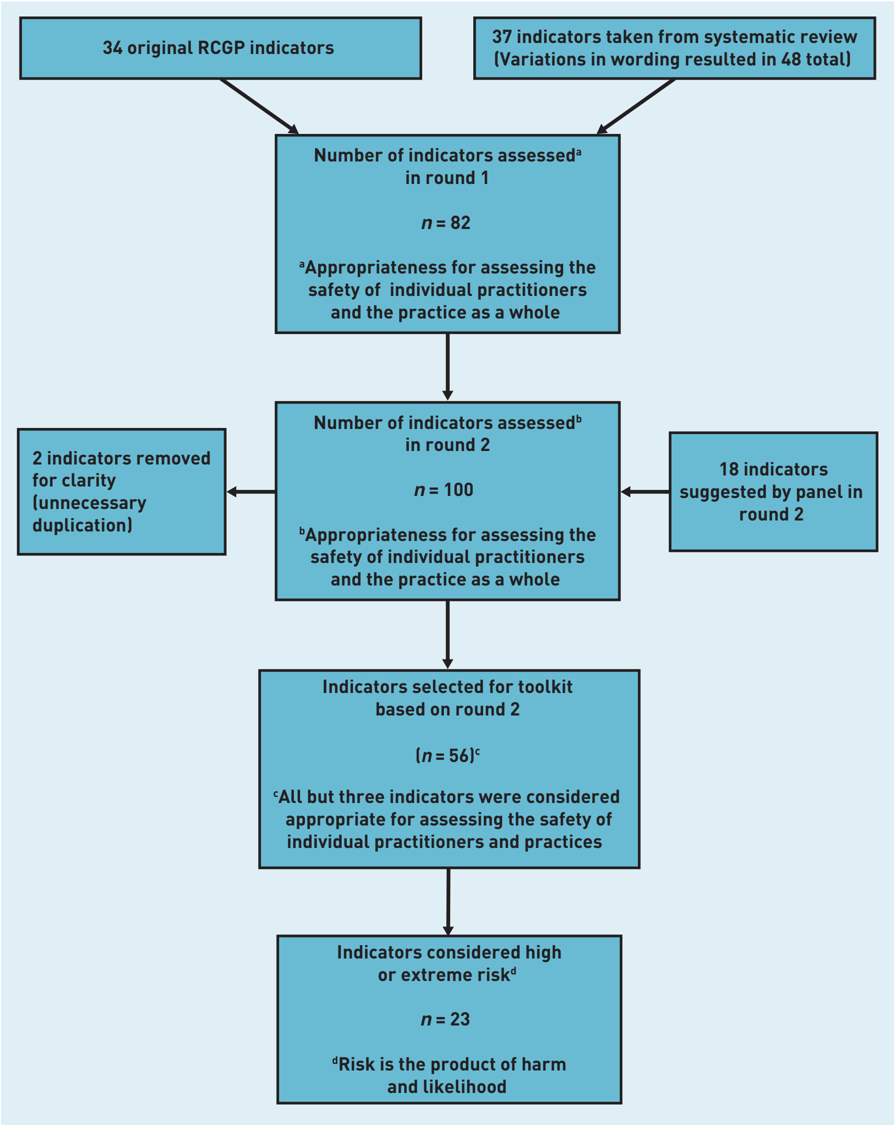

Figure 1. Number of indicators at each step of the consensus process.

at the final set of indicators and the criteria that were applied to select the indicators at each step. One hundred indicators were considered in round 2 of the exercise but two of these were removed because of unnecessary duplication; the results from the rating exercise are available from the authors on request. A total of 42 indicators were not included in the set; of these, 11 indicators were given a panel median rating of 1–3, 25 were given a rating of 4–6, and 6 achieved a median of 7–9, but there was a lack of consensus among the raters (as defined above). Therefore, 56 prescribing safety indicators were included in the final set as they were considered appropriate for assessing the safety of prescribing of individual GPs and/or general practices (overall panel median rating of 7–9, with agreement). These indicators are shown in Table 2 (further details are available from the authors). All but three of these indicators were considered appropriate for assessing the safety of individual GPs and general practices. Of the 56 indicators considered appropriate, five were suggested by the panel in round 2. Thirty-one of the 34 existing RCGP indicators were ratified for inclusion; the remaining 25 indicators were newly sourced.

Of the 56 indicators in Table 2, 19 were in the high risk category and four were in the extreme risk category, with 80% or more of the participants rating these items as either high risk or extreme risk. High risk and extreme risk indicators are fairly evenly distributed across the major categories. The median harm, likelihood, and risk scores for all 56 indicators and the percentage of responders who provided a score in the same risk category as the median are available from the authors on request.

DISCUSSION

Summary

Fifty-six prescribing-safety indicators were identified as appropriate for use in general practice. Violation of any of these indicators suggests a potential patient safety problem. These indicators covered hazardous prescribing across a range of therapeutic indications, hazardous drug–drug combinations, and inadequate laboratory test monitoring. Twenty-three of these indicators posed a high or extreme risk of harm to the patient.

Strengths and limitations

The strengths of this study include the wide range of sources and use of a formal literature review to identify prescribing-safety indicators. A large number of indicators (over 600) were reviewed and each indicator was presented to the panel accompanied by an evidence-based summary written by a clinical pharmacist. To construct the evidence-based summaries electronic searches of the literature were conducted and respected reference sources were drawn on.[22–24] This study also adhered to a validated systematic consensus method for developing appropriateness scenarios. Consistency in ratings was achieved by successfully recruiting most (9 out of 12) of the panel members who had originally rated the 2009 RCGP indicators.[15] There was also overlap (9 out of 11) between the panel members who provided the risk ratings and those who participated in the RAND exercise. In general, the method appears to be highly reliable as 31 of the 34 indicators that were

Table 2. Indicators rated as appropriate for assessing the safety of prescribing in general practice

Indicator	Risk of harm (1 = low, 2 = moderate, 3 = high, 4 = extreme)
**A: Cardiovascular and respiratory disease**	
1. Aspirin or clopidogrel prescribed to people with previous peptic ulcer or gastrointestinal bleed without gastroprotection[a]	3
2. Prescription of aspirin at a dose >75mg daily for ≥1 month in a patient aged >65 years	2
3. Prescription of digoxin at a dose >125 μg daily in a patient with renal impairment (for example, CKD 3 or worse)	3
4. Prescription of digoxin at a dose of greater than 125 μg daily for a patient with heart failure who is in sinus rhythm	3
5. Prescription of diltiazem or verapamil in a patient with heart failure[a]	3
6. Prescription of a beta-blocker to a patient with asthma (excluding patients who also have a cardiac condition, where the benefits of beta-blockers may outweigh the risks)[a]	3
7. Prescription of a long-acting beta-2 agonist inhaler to a patient with asthma who is not also prescribed an inhaled corticosteroid	3
**B: Central nervous system (including analgesics)**	
8. Prescription of a benzodiazepine or Z drug for ≥21 days, in a patient aged >65 years, who is not receiving benzodiazepines or Z drugs on a long-term basis	3
9. Initiation of prescription of benzodiazepine or Z drugs for ≥21 days in a patient aged >65 years with depression	3
10. Antipsychotics prescribed for >6 weeks in the over 65s with dementia but not psychosis[a]	3
11. Amitriptyline at dose >75mg prescribed to a patient with heart failure, arrhythmia, heart block. or postural hypotension	3
12. Prescription of aspirin to a child aged ≤16 years	2
13. Bupropion prescribed to a patient with epilepsy	3
**C: Anti-infective agents**	
14. Prescription of mefloquine to a patient with a history of convulsions[a]	3
**D: Endocrine and metabolic**	
15. Glitazone prescribed to patient with heart failure[a]	3
16. Metformin prescribed to a patient with renal impairment where the eGFR is ≤30ml/min[a]	4
17. Oral prednisolone prescribed at a dose ≥7.5mg daily for more than 3 months to the over 65s without co-prescription of osteoporosis-preventing treatments[a]	3
18. Modified-release potassium supplements prescribed to a patient with a history of peptic ulcer disease	2
**E: Women's health and urinary disorders**	
19. Prescription of a combined hormonal contraceptive to a woman with a history of venous or arterial thromboembolism	3
20. Prescription of oral or transdermal oestrogens to a woman with a history of breast cancer	3
21. Prescription of oral or transdermal oestrogen without a progestogen in a woman with an intact uterus	3
22. Prescription of a combined hormonal contraceptive to a woman aged ≥35 years who is a current smoker	3
23. Prescription of a combined hormonal contraceptive to a woman with a body mass index of ≥40[a]	3
**F: Immunosuppression**	
24. Methotrexate prescriptions should state 'weekly'	3
25. Methotrexate 2.5/10mg co-prescription	3
26. Methotrexate prescribed without folic acid	3
**G: Musculoskeletal**	
27. Concurrent use of two NSAIDS for more than 2 weeks (not including low-dose aspirin)	3
28. Prescription of an NSAID, without co-prescription of an ulcer-healing drug, to a patient with a history of peptic ulceration[a]	4
29. Prescription of an NSAID in a patient with heart failure[a]	3
30. Prescription of an NSAID in a patient with chronic renal failure with an eGFR <45[a]	4
31. Allopurinol prescribed at a dose of >200mg/day to patients with renal impairment (eGFR <30 or CKDA)[a]	3

... continued

Table 2 continued. Indicators rated as appropriate for assessing the safety of prescribing in general practice

Indicator	Risk of harm (1 = low, 2 = moderate, 3 = high, 4 = extreme)
**H: Hazardous co-prescriptions and allergy**	
32. Prescription of warfarin and aspirin in combination (without co-prescription of gastroprotection)[a]	3
33. Concurrent use of warfarin and any antibiotic without monitoring the INR within 5 days[a,b]	4
34. Prescription of warfarin in combination with an oral NSAID	3
35. Prescription of a phosphodiesterase type-5 inhibitor, for example sildenafil, to a patient who is also receiving a nitrate or nicorandil[a,c]	3
36. Co-prescription of lithium with thiazide diuretic	3
37. Prescription of a potassium salt or potassium-sparing diuretic (excluding aldosterone antagonists) to a patient who is also receiving an ACE inhibitor or angiotensin II receptor antagonist[a]	3
38. Prescription of verapamil to a patient who is also receiving a beta-blocker	3
39. Co-prescription of itraconazole with simvastatin, or with atorvastatin at a dose ≥80mg[a,c]	3
40. Co-prescription of trimethoprim with methotrexate for >7 days[a]	3
41. Prescription of clarithromycin or erythromycin to a patient who is also receiving simvastatin, with no evidence that the patient has been advised to stop the simvastatin while taking the antibiotic[a]	3
42. Prescription of a penicillin-containing preparation to a patient with a history of allergy to penicillin	4
**I: Laboratory test monitoring**	
43. Patients aged >75 years on loop diuretics who have not had a U+E in the previous 15 months[a]	3
44. Prescription of amiodarone without a record of liver function being measured in the previous 9 months	3
45. Prescription of amiodarone without a record of thyroid function being measured within the previous 9 months	3
46. Prescription of an ACE inhibitor or angiotensin II receptor antagonist without a record of renal function and electrolytes being measured prior to starting therapy	3
47. Patients on an ACE inhibitor or angiotensin II receptor antagonist who have not had a U+E in the previous 15 months[a]	3
48. Prescription of warfarin to a patient without a record of INR having been measured within the previous 12 weeks (excluding patients who self-monitor)	4
49. Prescription of a statin without an ALT taken prior to starting treatment	3
50. Prescription of a statin without an ALT taken prior to starting treatment and within 3 months of starting treatment	3
51. Prescription of lithium without a record of a lithium level being measured within the previous 6 months[a]	3
52. Metformin without yearly serum creatinine	3
53. Use of a hypothyroid agent without monitoring relevant thyroid function tests within 2–4 months of initiation or dosage change and at least every 15 months thereafter	3
54. Prescription of methotrexate without a record of a full blood count within the previous 3 months	3
55. Prescription of methotrexate without a record of liver function having been measured within the previous 3 months	3
56. Allopurinol without baseline urea, electrolytes, creatinine and eGFR	2

[a]For 23 items (19 high risk and 4 extreme risk) 80% or more of the responders rated the indicator as high or extreme risk. [b]Consensus reached for assessing the safety of prescribing of practices, but not individual GPs. [c]Consensus reached for assessing the safety of prescribing of individual GPs, but not practices. ACE inhibitor = angiotensin-converting enzyme inhibitor. ALT = alanine transferase. CKD = chronic kidney disease. eGFR = estimated glomerular filtration rate. INR = International Normalised Ratio. NSAID = non-steroidal anti-inflammatory drug. U+E = urea and electrolytes.

previously presented in the 2009 RAND Appropriateness Method exercise were still given ratings high enough for inclusion. Three indicators from the earlier set were not included because they received ratings of 6–7. The degree of risk of harm to patients associated with each indicator was assessed and this is potentially helpful in terms of identifying which indicators may be most important to focus on when trying to identify patients at greatest risk within a general practice. However, a limitation is that the views of the GP panel members were sought just once in relation to this aspect of the study.

Comparison with existing literature

New indicators found in the systematic review process were added to an existing set of 34 indicators previously published by

the authors,[15] to update the set. Owing to constant updates in the field of prescribing indicators, a large set of indicators have been published by a team in Scotland since the authors' systematic review was undertaken and these will be considered in future work.[30] In contrast to a 2013 study[29] of secondary care prescribing indicators (in which 80 out of 109 indicators (73%) were considered high or extreme risk) a smaller proportion were considered high or extreme risk (23 out of 56 or 41%) in this study. This is likely to be due to differences in the indicators and differences in risk of harm between primary and secondary care.

Implications for research and practice

The results from this study have implications for future research and practice. The authors plan to test the acceptability, feasibility, reliability, and validity of the prescribing indicators in a sample of English general practices. This will aim to determine whether there are any problems with implementing the indicators in general practices and will enable the description of variations in the safety of prescribing between practices. An intervention study will also be conducted to determine whether these indicators, as part of a larger patient safety toolkit, can be used to improve patient safety. The indicators might equally well be used for practice learning, revalidation or audit purposes (see article series in *Prescriber*[31–36] for examples of how this can be accomplished).

Work will also be conducted to determine whether each indicator can be translated into a computer query capable of assessing the prescribing safety of individual GPs. The use of these queries is being tested on GP computer systems to examine whether the use of computerised prescribing-safety indicators improves prescribing performance. The challenge for future work would be to prove that this does improve patient outcomes. This goal has already been achieved in the PINCER trial[16] for several of the indicators.

Funding

National Institute for Health Research School for Primary Care Research, Project Number 113.

Provenance

Freely submitted; externally peer reviewed.

Competing interests

The authors have declared no competing interests.

Acknowledgements

We wish to thank the following GPs who participated in rating the indicators: Olesya Atkinson, William Beebe, Beth Coward, Maureen Crawford, Martin Duerdens, Simon Hurding, Adam Liew, Rosalind Mills, Julie Osborne, Lindsay Pope, Beth Rimmer, Mohammed Sharif, Bijoy Sinha, and Arnold Zermansky.

Discuss this article

Contribute and read comments about this article: **www.bjgp.org/letters**

REFERENCES

1. Health and Social Care Information Centre. *Prescriptions dispensed in the community, statistics for England: 2001 to 2011*. 2012. http://www.hscic.gov.uk/catalogue/PUB06941 (accessed 22 Jan 2014).
2. Howard R, Avery A, Bissell P. Causes of preventable drug-related hospital admissions: a qualitative study. *Qual Saf Health Care* 2008; **17(2):** 109–116.
3. Fick DM, Cooper JW, Wade WE, *et al*. Updating the Beers criteria for potentially inappropriate medication use in older adults: results of a US consensus panel of experts. *Arch Intern Med* 2003; **163(22):** 2716–2724.
4. Wenger NS, Shekelle PG. Assessing care of vulnerable elders: ACOVE project overview. *Ann Intern Med* 2001; **135(8 Pt 2):** 642–646.
5. Gallagher P, Ryan C, Byrne S, *et al*. STOPP (Screening Tool of Older Person's Prescriptions) and START (Screening Tool to Alert doctors to Right Treatment). Consensus validation. *Int J Clin Pharmacol Ther* 2008; **46(2):** 72–83.
6. Wessell AM, Litvin C, Jenkins RG, *et al*. Medication prescribing and monitoring errors in primary care: a report from the Practice Partner Research Network. *Qual Saf Health Care* 2010; **19(5):** e21.
7. Rognstad S, Brekke M, Fetveit A, *et al*. The Norwegian General Practice (NORGEP) criteria for assessing potentially inappropriate prescriptions to elderly patients. A modified Delphi study. *Scand J Prim Health Care* 2009; **27(3):** 153–159.
8. Basger BJ, Chen TF, Moles RJ. Inappropriate medication use and prescribing indicators in elderly Australians: development of a prescribing indicators tool. *Drugs Aging* 2008; **25(9):** 777–793.
9. Raebel MA, Chester EE, Newsom EE, *et al*. Randomized trial to improve laboratory safety monitoring of ongoing drug therapy in ambulatory patients. *Pharmacotherapy* 2006; **26(5):** 619–626.
10. Zhan C, Correa-de-Araujo R, Bierman AS, *et al*. Suboptimal prescribing in elderly outpatients: potentially harmful drug-drug and drug-disease combinations. *J Am Geriatr Soc* 2005; **53(2):** 262–267.
11. McLeod PJ, Huang AR, Tamblyn RM, Gayton DC. Defining inappropriate practices in prescribing for elderly people: a national consensus panel. *CMAJ* 1997; **156(3):** 385–391.
12. Avery AJ, Heron T, Lloyd D, *et al*. Investigating relationships between a range of potential indicators of general practice prescribing: an observational study. *J Clin Pharm Ther* 1998; **23(6):** 441–450.
13. Campbell SM, Cantrill JA, Roberts D. Prescribing indicators for UK general practice: Delphi consultation study. *BMJ* 2000; **321:** 425–428.
14. Morris CJ, Rodgers S, Hammersley VS, *et al*. Indicators for preventable drug related morbidity: application in primary care. *Qual Saf Health Care* 2004; **13(3):** 181–185.
15. Avery AJ, Dex GM, Mulvaney C, *et al*. Development of prescribing-safety indicators for GPs using the RAND Appropriateness Method. *Br J Gen Pract* 2011; DOI: 10.3399/bjgp11X588501.
16. Avery AJ, Rodgers S, Cantrill JA, *et al*. A pharmacist-led information technology intervention for medication errors (PINCER): a multicentre, cluster randomised, controlled trial and cost-effectiveness analysis. *Lancet* 2012; **379:** 1310–1319.
17. Guthrie B, McCowan C, Davey P, *et al*. High risk prescribing in primary care patients particularly vulnerable to adverse drug events: cross sectional population database analysis in Scottish general practice. *BMJ* 2011; **342:** d3514.
18. Williams D, Bennett K, Feely J. The application of prescribing indicators to a primary care prescription database in Ireland. *Eur J Clin Pharmacol* 2005; **61(2):** 127–133.
19. Bregnhøj L, Thirstrup S, Kristensen MB, Sonne J. Reliability of a modified medication appropriateness index in primary care. *Eur J Clin Pharmacol* 2005; **61(10):** 769–773.
20. Hammersley VS, Morris CJ, Rodgers S, *et al*. Applying preventable drug-related morbidity indicators to the electronic patient record in UK primary care: methodological development. *J Clin Pharm Ther* 2006; **31(3):** 223–229.
21. Higgins JPT, Green S (Eds). *Cochrane handbook for systematic reviews of interventions*. Version 5.1.0. The Cochrane Collaboration, 2011. http://www.cochrane-handbook.org/ (accessed 22 Jan 2014).
22. Joint Formulary Committee. *British national formulary*. 63rd ed. London: BMJ Group and RPS Publishing, 2012.
23. Baxter K, ed. *Stockley's drug interactions*. 9th edn. London: Royal Pharmaceutical Society of Great Britain; Pharmaceutical Press, 2010.
24. Sweetman SC, ed. *Martindale: the complete drug reference*. 37th edn. London: Pharmaceutical Press, 2011.
25. RAND Health. *RAND/UCLA appropriateness method*. Santa Monica, CA: RAND, 2001. http://www.rand.org/health/surveys_tools/appropriateness.html (accessed 22 Jan 2014).
26. NHS Institute for Innovation and Improvement. *The good indicators guide: understanding how to use and choose indicators*. Coventry: NHS Institute for Innovation and Improvement, 2008.
27. Shekelle PG. Appropriateness criteria: a useful tool for the cardiologist. *Heart* 2009; **95(7):** 517–520.
28. Campbell SM, Braspenning J, Hutchinson A, Marshall M. Research methods used in developing and applying quality indicators in primary care. *Qual Saf Health Care* 2002; **11(4):** 358–364.
29. Thomas SK, McDowell SE, Hodson J, *et al*. Developing consensus on hospital prescribing indicators of potential harms amenable to decision support. *Br J Clin Pharmacol* 2013; **76(5):** 797–809.
30. Dreischulte T, Grant AM, McCowan C, *et al*. Quality and safety of medication use in primary care: consensus validation of a new set of explicit medication assessment criteria and prioritisation of topics for improvement. *BMC Clin Pharmacol* 2012; **12:** 5.
31. Spencer R, Serumaga B. Prescribing long-acting beta2-agonist inhalers in asthma. *Prescriber* 2011; **22(15–16):** 26–28. DOI: 10.1002/psb.784.
32. Spencer R, Serumaga B. Concurrent macrolide and statin: a common interaction. *Prescriber* 2011; **22(17):** 49–50. DOI: 10.1002/psb.796.
33. Spencer R, Serumaga B. Prescribing antiemetics for patients with Parkinson's. *Prescriber* 2011; **22(18):** 48–49. DOI: 10.1002/psb.804.
34. Spencer R, Serumaga B. Co-prescription of a nitrate or nicorandil with a PDE-5 inhibitor. *Prescriber* 2011; **22(19):** 71–72. DOI: 10.1002/psb.816.
35. Spencer R, Serumaga B. Digoxin doses over 125µg daily in vulnerable patient groups. *Prescriber* 2011; **22(22):** 34–35. DOI: 10.1002/psb.841.
36. Spencer R, Serumaga B. Beta-blockers in patients with asthma and COPD. *Prescriber* 2012; **23(4):** 14–16. DOI: 10.1002/psb.866.

Appendix 1. Literature review search strategy

Setting

"Family physician" OR "primary care" OR "family practice" OR "general practice" OR "ambulatory care" OR "ambulatory health" OR "ambulatory health-care" OR "ambulatory healthcare" OR "community health" OR "community healthcare" OR "community health-care" OR "primary health" OR "primary healthcare" OR "primary physician" OR "primary health-care" OR generalist OR "family medicine"

Safety synonyms

"administration error" OR "administration errors" OR "dispensing error" OR "dispensing errors" OR "medication error" OR "medication errors" OR "medical mistake" OR "medical mistakes" OR "prescription error" OR "prescription errors" OR "prescribing error" OR "prescribing errors" OR " prescribing fault" OR "prescribing faults" OR "medical error" OR "medical errors" OR malpractice OR safety OR "safety-culture" OR "adverse event" OR "adverse events" OR "adverse effect" OR "adverse effects" OR "adverse reaction" OR "adverse reactions" OR harm OR harms

Types of tools

"scale" OR "scales" OR "survey" OR "surveys" OR "questionnaire" OR "questionnaires" OR "instrument" OR "instruments" OR "indicator" OR "indicators" OR "outcome assessment" OR "outcome assessments" OR "patient reported outcome" OR "patient reported outcomes" OR "patient experience" OR "patient experiences" OR "practice guideline" OR "practice guidelines" OR "quality assurance" OR tool OR tools OR toolkit OR toolkits
