# Supplementary material for: Exploring the challenges to safer prescribing and medication monitoring in prisons: A qualitative study with health care staff
Source: PLoS One. 2022 Nov 3;17(11):e0275907. doi: 10.1371/journal.pone.0275907 (PMC9632766; doi:10.1371/journal.pone.0275907)
Supplement: S4 File — (ZIP) [file pone.0275907.s004.zip › Supplementary file - Harms/Supp file 7.2 Paper 2 Pre-reading Thomas - 2013.pdf]

BJCP British Journal of Clinical Pharmacology

DOI:10.1111/bcp.12087

Developing consensus on hospital prescribing indicators of potential harms amenable to decision support

Sarah K. Thomas,[1] Sarah E. McDowell,[2] James Hodson,[2] Ugochi Nwulu,[2] Rachel L. Howard,[3] Anthony J. Avery,[4] Ann Slee[1] & Jamie J. Coleman[1,2]*

[1]School of Clinical and Experimental Medicine, University of Birmingham, [2]University Hospitals Birmingham NHS Foundation Trust, Birmingham, [3]School of Pharmacy, University of Reading, Reading and [4]School of Community Health Sciences, University of Nottingham, Nottingham, UK

Correspondence
Dr Jamie J. Coleman MD, MA, MRCP(UK), College of Medical and Dental Sciences, University of Birmingham, Edgbaston, Birmingham B15 2TT, UK.
Tel.: +44 121 371 6003
Fax: +44 121 371 4204
E-mail: j.j.coleman@bham.ac.uk

*On behalf of the ePrescribing Programme Team: Aziz Sheikh, Tony Avery, Antony Chuter, Kathrin Cresswell, David W. Bates, Alan Girling, Richard Lilford, Zoe Morrison, Rosemary Porteous, Ann Robertson, Jill Schofield, Jonathan Shapiro, Ann Slee, Sarah Slight, Robin Williams, Jianhua Wu, Guiqing Lily Yao and Shihua Zhu.

Keywords
clinical decision support, consensus, drug prescriptions, hospitals, medication errors, quality indicators

Received
4 December 2012

Accepted
17 January 2013

Accepted Article Published Online
31 January 2013

WHAT IS ALREADY KNOWN ABOUT THIS SUBJECT

- Untargeted prescription chart review for prescribing errors can lead to a plethora of low- or no-harm errors.
- Prescribing indicators are a valid method to measure or monitor an area of prescribing.
- At present, there is no validated list of indicators for the hospital setting.

WHAT THIS STUDY ADDS

- Eighty prescribing indicators have been identified with a high potential for causing patient harm in the hospital setting.
- Use of the indicators can standardize data collection for high-risk errors.
- The indicators are amenable to clinical decision support, so can assist pre/post implementation studies of such technology.

AIMS

To develop a list of prescribing indicators specific for the hospital setting that would facilitate the prospective collection of high-severity and/or high-frequency prescribing errors, which are also amenable to electronic clinical decision support.

METHODS

A two-stage consensus technique (electronic Delphi) was carried out with 20 experts across England. Participants were asked to score prescribing errors using a five-point Likert scale for their likelihood of occurrence and the severity of the most likely outcome. These were combined to produce risk scores, from which median scores were calculated for each indicator across the participants in the study. The degree of consensus between the participants was defined as the proportion that gave a risk score in the same category as the median. Indicators were included if a consensus of 80% or more was achieved.

RESULTS

A total of 80 prescribing errors were identified by consensus as being high or extreme risk. The most common drug classes named within the indicators were antibiotics ($n = 13$), antidepressants ($n = 8$), nonsteroidal anti-inflammatory drugs ($n = 6$) and opioid analgesics ($n = 6$). The most frequent error type identified as high or extreme risk were those classified as clinical contraindications ($n = 29$ of 80).

CONCLUSIONS

Eighty high-risk prescribing errors in the hospital setting have been identified by an expert panel. These indicators can serve as a standardized, validated tool for the collection of prescribing data in both paper-based and electronic prescribing processes. This can assess the impact of safety improvement initiatives, such as the implementation of electronic clinical decision support.

© 2013 The Authors
British Journal of Clinical Pharmacology © 2013 The British Pharmacological Society

Introduction

Medication errors are relatively common in hospital practice and can lead to preventable patient harm [1]. Monitoring the types and rates of errors is crucial in understanding how processes can be improved to reduce the risk of patient harm, and to examine whether an intervention to improve a process has had the desired impact. Determining the potential harm of these errors and the subsequent burden to both the patient and the National Health Service (NHS) can prove difficult. Indeed many studies choose to utilize their own severity scales for defining a level of harm. These tend to be subjectively assessed and scored by the researcher and therefore introduce a degree of bias, or request consensus between a number of healthcare professionals for every error found in the research [2, 3], which can prove time consuming and costly.

Untargeted prescription chart review for potential prescribing errors can lead to a plethora of low- or no-harm errors [3, 4]. Whilst newer processes exist for determining 'actual' harm occurrences – for example, by looking at triggers that indicate harms such as the prescribing of antidote drugs or critical laboratory values [5] – such processes require an intensive retrospective review of care records. Determining the preventable nature of such harm is also prone to subjective interpretation.

Prescribing indicators are agreed by a range of stakeholders to be a valid method to measure or monitor an area of prescribing, where there is a perceived direction in which the prescribing being measured should move over time [6]. Previous work in general practice has identified a list of critical indicators of potential prescribing errors in the UK as a means of assessing the safety of general practitioner (GP) prescribing [7]. In a similar manner, inappropriate prescribing (IP) criteria for older adults have been developed to facilitate chart review and identify the medications that may 'potentially' lead to adverse drug events [8, 9]. However, this 'screening' tool is restricted to errors of omission and commission, is specific to a patient population, and is not necessarily designed to measure prescribing over time. In Australia, hospitals are encouraged to use the 'Indicators for Quality Use of Medicines (QUM)', which is a set of 30 indicators designed to measure both processes and outcomes of medication use to inform system improvement [10]. These indicators are generally not drug specific and capture data on quality rather than safety, looking, for example, at indicators for optimal medication use rather than indicators of potential harms. At present, therefore, there remains no validated list of 'prescribing' indicators that have been developed for the hospital setting or that are associated with both the highest risk of patient harm and likelihood of occurrence.

As Computerized Physician Order Entry (CPOE) becomes more widespread in the hospital setting, prescribing indicators should be amenable to being addressed by clinical decision support (CDS); that is, the error to which each indicator refers has the potential to be prevented by such software. Clinical decision support provides an opportunity to alert prescribers to potential harms [11–13]. Such systems have been shown to reduce medication error rates substantially, but most studies have not been powered to detect differences in the rate of adverse drug events [12]. The methods adopted by the researchers and the study outcomes also vary, making comparisons between them difficult. The development of indicators that are amenable to CDS allows for the effects of this technology to be quantified, which is not only important given the rate at which such technologies are being implemented but also relevant because of the heterogeneity of system configuration and complexity.

The aim of this research was to produce a list of prescribing indicators specific for the hospital setting that would aid the prospective collection of high-severity and/or high-frequency prescribing error data, in both paper-based and electronic medication processes.

Methods

The Delphi technique has been widely used in healthcare research as an approach to establish consensus in an area where published information is inadequate [8, 14]. This multistage methodology was selected to gather the subjective judgements of experts to develop and finalize a list of hospital prescribing indicators. We set out to complete an exploratory round, followed by two rounds of an eDelphi to identify high- or extreme-risk indicators with a consensus of at least 80% across an expert panel (Figure 1).

Expert panel selection

Members of the research team (a pharmacist, clinical pharmacologist, epidemiologist and senior researcher in public health) selected participants based on their clinical expertise in medication safety, as well as those with knowledge of CPOE systems who would be able to identify errors that are amenable to reduction by CDS software. Some participants were recruited from a National Electronic Prescribing Conference (carried out as part of a Programme Grant to investigate prescribing safety in hospital ePrescribing systems) [15], as well as personal contacts of the research team.

A total of 32 experts were invited to participate in the process, of whom 20 agreed. Panellists were pharmacists, clinical pharmacologists and physicians from geographically diverse areas in England, with a range of professional grades.

The eDelphi process

In the first instance, the research team defined inclusion and exclusion criteria for the prescribing indicators (see

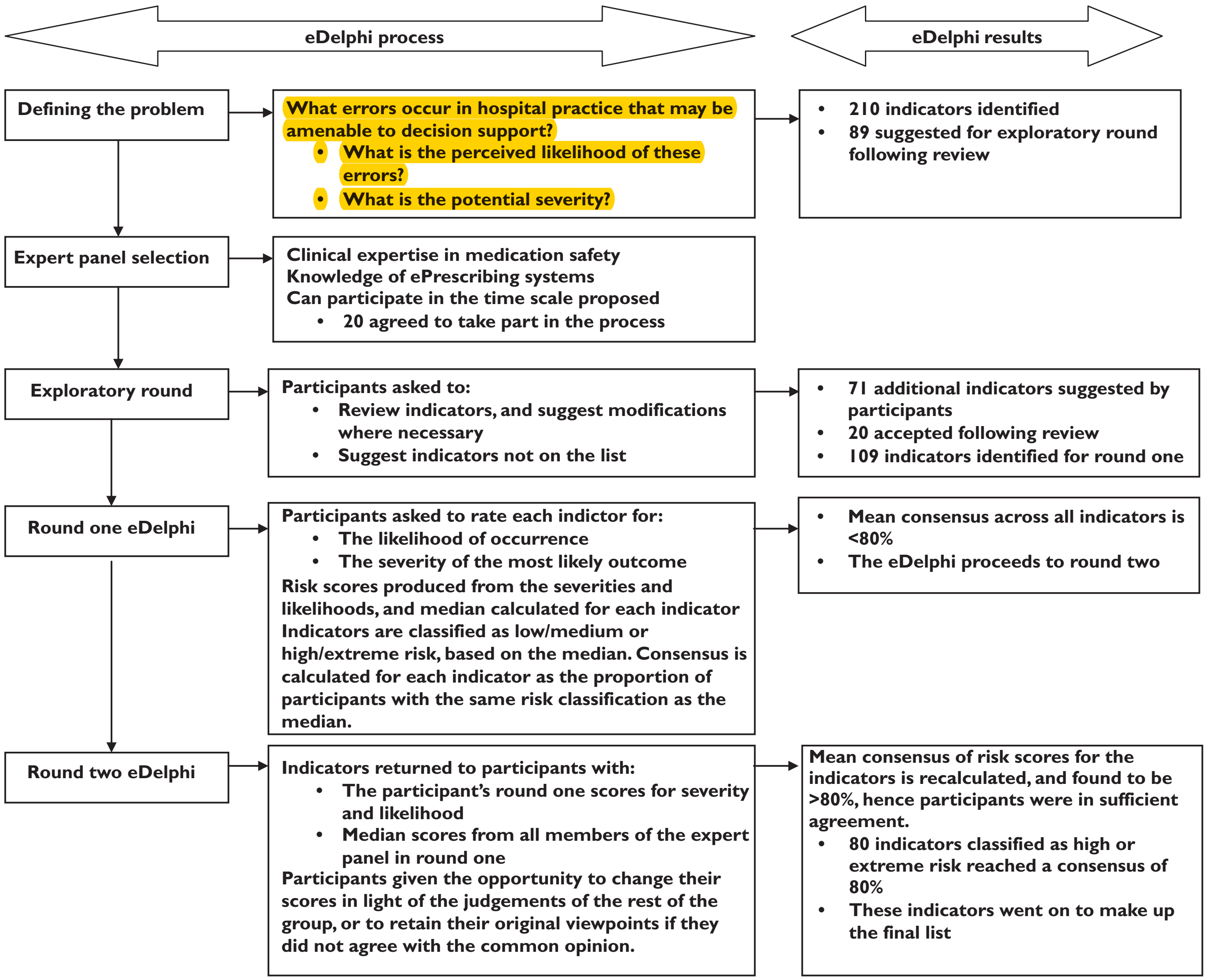

Figure 1

A summary of the eDelphi process and results

Table 1). These criteria were used to construct an initial list of indicators based on clinical experience, searches of relevant UK resources and previous work conducted to define critical indicators of potential harm [8, 16–20]. Where possible, the original evidence base or language used by others to describe the issues was adopted to provide the context of the indicators. Each indicator was developed to state the trigger drug(s), the error process and the associated harm, in order to reduce the risk of misunderstanding and bias; for example, 'digoxin [drug] prescribed concomitantly [process] with a diuretic [drug] (risk of hypokalaemia and subsequent digoxin toxicity [harm])'. The prescribing indicators were summarized in a table and then circulated among the research team for comments and refinement before incorporation into a questionnaire for circulation to the participants for an exploratory round.

Exploratory round The refined list of indicators was emailed to all participants enrolled in the study. They were asked to review each of the indicators and to recommend any modifications they deemed necessary. In addition, the opportunity was given for further indicators to be suggested that participants felt were missing from the initial list. The responses from this round were assessed by the research team, and those which had clinical merit were included in an updated list of indicators for round one of the eDelphi. A rationale for excluding suggested indicators was provided to each of the participants to give a clearer understanding of the overall inclusion criteria. For example, one participant suggested 'any use of naloxone or flumazenil'. The rationale provided to the participant stated 'this is a trigger to identify an adverse drug event, not an indicator of harm from a prescribing error'.

Table 1

Inclusion and exclusion criteria for the prescribing indicators

Inclusion criteria:
• The indicator describes a drug prescribed in the general adult in-patient population
• The indicator relates to a drug prescribed at a reasonable rate in the UK hospital environment
Exclusion criteria:
• The indicator describes a prescribing practice that is not routinely undertaken in the UK hospital setting
• The indicator is specific to a drug used in a patient population other than adult in-patients (i.e. paediatric vaccination schedules)
• The indicator describes an error which would not be amenable to clinical decision support
• Extraction of data required for the indicator (from hospital care records) is unlikely to be feasible
• The indicator describes a failure to monitor treatment
• The indicator describes errors relating to the dispensing or administration of a drug

Table 2

Scoring likelihood and severity of the errors occurring (from the UK National Patient Safety Agency Risk Matrix [21])

Consequence	Likelihood<br>1 Rare<br>This will probably never occur	2 Unlikely<br>Do not expect it to occur, but it is possible it may do	3 Possible<br>This might occasionally occur	4 Likely<br>This will probably occur	5 Almost certain<br>This will undoubtedly occur, possibly frequently
**5 Catastrophic**<br>Leads to death, multiple permanent injuries or irreversible health effects	5	10	15	20	25
**4 Major**<br>Major injury, leading to long-term incapacity/disability	4	8	12	16	20
**3 Moderate**<br>Moderate injury, requiring intervention	3	6	9	12	15
**2 Minor**<br>Minor injury or illness, requiring minor intervention	2	4	6	8	10
**1 Insignificant**<br>No risk of patient injury or harm and no intervention required	1	2	3	4	5

1–3	4–6	8–12	15–25
Low risk	Moderate risk	High risk	Extreme risk

Round one Round one of the eDelphi aimed to identify the most clinically significant indicators, defined as those which would have the greatest risk in a clinical setting. Using a five-point Likert scale, participants were asked to rank each indicator for the likelihood of it occurring in hospital practice, and the severity of the most likely outcome should the error occur. This scale was based on that used by the UK National Patient Safety Agency, National Reporting and Learning Service (NRLS; see Table 2) [21], and therefore one with which UK healthcare professionals were likely to be familiar. Acknowledging that participants' previous areas of clinical practice (e.g. oncology) may influence the scoring, they were requested to take a more general view in the interpretation of each indicator.

When all ratings from round one had been received, the likelihood and severity scores were converted into 'risk scores', using the NRLS Risk Matrix (Table 2) [21]. The median scores for each indicator were then calculated across the participants in the study, and the indicators divided into the following two groups: those where the median risk score was situated in the upper categories of 'high' or 'extreme', and those where the risk was 'low' or 'moderate'. The degree of consensus between the participants was defined as the proportion that gave a risk score in the same group as the median. The mean consensus across all of the indicators was then calculated. The target for consensus was defined as at least 80%, in order to ensure that the resulting list of indicators was reliable. This adhered to validated consensus method for developing appropriateness scenarios [22].

Round two In round two, the full list of indicators was returned to each participant, with their own individual scores for severity and likelihood shown, as well as the median scores from all members of the expert panel. This gave the opportunity for participants to modify their scores in light of the judgements of the rest of the group, or to retain their original viewpoints if they did not agree with the common opinion. The median risk scores were

Table 3

Demographic details of the 20 participants who took part in the eDelphi process

Profession	Grade	Specialty	Employer
**Pharmacist**	Senior	Diabetes and endocrinology	NHS hospital
**Doctor**	Registrar	Clinical pharmacology	Academic institution
**Doctor**	FY2	Medicine	NHS hospital
**Doctor**	Consultant	Clinical pharmacology	NHS hospital
**Pharmacist**	Teacher practitioner	Paediatrics	NHS hospital
**Pharmacist**	Senior	General surgery	NHS hospital
**Pharmacist**	Lecturer	Palliative care	NHS hospital
**Pharmacist**	Lecturer	Medication safety	Academic institution
**Pharmacist**	Senior	Oncology	NHS hospital
**Pharmacist**	Lecturer	Medication safety	Academic institution
**Pharmacist**	Lead pharmacist/lecturer	Medication safety	Academic institution
**Pharmacist**	Senior	Electronic prescribing	NHS hospital
**Doctor**	FY2	General medicine	NHS hospital
**Pharmacist**	Senior	Primary care	Interface*
**Doctor**	FY2	Medicine	NHS hospital
**Doctor**	Registrar	Respiratory medicine	NHS hospital
**Doctor**	FY1	Medicine	NHS hospital
**Doctor**	Registrar	Clinical pharmacology	NHS hospital
**Doctor**	FY2	Diabetes and endocrinology	NHS hospital
**Pharmacist**	Lecturer	Pharmacy practice	Academic institution

*Works at the interface between primary and secondary care. Abbreviations are as follows: FY1, Foundation Year 1 doctor (first year of practice postqualification); FY2, Foundation Year 2 doctor (second year of practice postqualification); and NHS, National Health Service.

then recalculated, and the mean consensus between participants was determined, as for round one. The final list of indicators contained indicators where the median risk score and the scores of at least 80% of participants were in the 'high' (risk score 3) or 'extreme' (risk score 4) categories.

Results

The exploratory stage and two-round eDelphi were completed by all 20 participants. The expert panel consisted of 11 pharmacists with a sum of 122 years of hospital experience and nine physicians with a sum of 60 years of hospital experience (Table 3). All participants either worked in an academic institution or within an NHS hospital, and all had an interest in medication safety and/or electronic prescribing.

In the first instance, 210 prescribing indicators were identified; 108 of these were from published studies using similar consensus techniques [8, 16, 17], 36 from safety warnings and alerts from UK authorities [18–20] and 66 from clinical experience.

In the exploratory round, a refined list of 89 indicators was sent to the participants, and 71 additional prescribing indicators were suggested by the expert panel, of which 20 were selected for inclusion in round two (making a total of 109). The rationale for excluding 50 of the additional indicators is given in Table 4.

A total of 80 of 109 prescribing errors were considered high or extreme risk by consensus when the scores for likelihood and severity were considered; these were included in the final screening tool (see Table 5). The indicators excluded in round two are summarized in Table 6.

Table 4

Reasons for exclusion of suggested indicators in round one of the eDelphi

Reason for exclusion	Number
**Modified existing indicator**	2
**Dependent on individual hospital guidelines**	1
**Difficult to assess**	9
**Drug not prescribed on an in-patient basis**	1
**Indicator already present in list**	10
**Not amenable to decision support**	6
**Specialty specific**	4
**Relates to administration**	8
**Relates to monitoring**	7
**Trigger, not indicator**	1
**Unnecessary duplication of treatment**	1
Total	50

Of the 80 final indicators, the majority were synthesized from clinical experience ($n = 25$), followed by those identified by the STOPP/START (a screening tool of potentially inappropriate medicines or omission of appropriate medicines in the older adult population) criteria [8] ($n = 23$), Avery *et al.* ($n = 12$) [16], Medicines and Healthcare products Regulatory Agency and National Patient Safety Agency (NPSA) warnings ($n = 14$) and Phansalkar *et al.* ($n = 6$) [17].

Table 5

High- and extreme-risk prescribing indicators, with median scores from the eDelphi shown for the severity and likelihood, with a calculated risk score

Prescribing safety indicator title	Group	Error type	Median scores Severity	Likelihood	Risk	Percentage agreement
**Low-molecular-weight heparin prescribed without the patient's weight being used to calculate the treatment dose *(risk of subtherapeutic or supratherapeutic dosing)***	Cardiovascular	Dosing	3	4	3	95%
**Low-molecular-weight heparin prescribed at a dose exceeding the maximum as stated in the product literature *(risk of bleeding increased)***	Cardiovascular	Dosing	4	3	3	90%
**Digoxin prescribed at a dose >125 μg daily to a patient with renal impairment *(increased risk of digoxin toxicity)***	Cardiovascular	Dosing	3	3	3	95%
**Digoxin prescribed at a dose of >125 μg daily to a patient with heart failure who is in sinus rhythm *(increased risk of digoxin toxicity)***	Cardiovascular	Dosing	3	3	3	95%
**Amiodarone prescribed to a patient with abnormal thyroid function tests *(increased risk of thyroid disorders)***	Cardiovascular	Clinical contraindication	3	3	3	80%
**Noncardioselective β-adrenoceptor-blocking drug prescribed to a patient with chronic obstructive pulmonary disease *(increased risk of bronchospasm)***	Cardiovascular	Clinical contraindication	3	3	3	85%
**Angiotensin-converting enzyme inhibitor or angiotensin II receptor antagonist prescribed to a patient with a potassium level ≥5.0 mmol l^{-1} *(can cause or exacerbate hyperkalaemia)***	Cardiovascular	Clinical contraindication	3	3	3	80%
**Verapamil prescribed to a patient with NYHA Class III or IV heart failure *(risk of precipitating heart failure, exacerbating conduction disorders and causing significant deterioration)***	Cardiovascular	Clinical contraindication	4	3	3	95%
**Low-molecular-weight heparin prescribed to a patient with renal impairment without dose adjustment *(increased risk of bleeding)***	Cardiovascular	Dosing	3	4	3	100%
**Warfarin prescribed to a patient with a concurrent bleeding disorder *(increased risk of bleeding)***	Cardiovascular	Clinical contraindication	4	2	3	100%
**Aspirin prescribed to a patient with a past medical history of peptic ulcer disease without antisecretory drugs or mucosal protectants *(increased risk of peptic ulceration and risk of bleeding)***	Cardiovascular	Clinical contraindication	4	3	3	95%
**Antiplatelet medication prescribed to a patient with a concurrent bleeding disorder *(increased risk of bleeding)***	Cardiovascular	Clinical contraindication	4	3	3	100%
**Digoxin prescribed concomitantly with a diuretic *(risk of hypokalaemia and subsequent digoxin toxicity)***	Cardiovascular	Drug–drug interaction	3	4	3	100%
**Statin prescribed concomitantly with a macrolide antibiotic *(increased risk of myopathy)***	Cardiovascular	Drug–drug interaction	3	4	3	95%
**Potassium-sparing diuretic (excluding aldosterone antagonists) prescribed to a patient also receiving an angiotensin-converting enzyme inhibitor or angiotensin II receptor antagonist *(increased risk of severe hyperkalaemia)***	Cardiovascular	Drug–drug interaction	3	3	3	90%
**Amiodarone prescribed concomitantly with simvastatin 40 mg or above *(increased risk of myopathy)***	Cardiovascular	Drug–drug interaction	3	3	3	85%
**Verapamil prescribed to a patient concomitantly a with β-adrenoceptor-blocking drug *(increased risk of adverse cardiovascular effects)***	Cardiovascular	Drug–drug interaction	4	3	3	90%
**Warfarin prescribed concomitantly with a nonsteroidal anti-inflammatory drug *(increased risk of bleeding)***	Cardiovascular	Drug–drug interaction	4	3	3	100%
**Clopidogrel prescribed to a patient concomitantly with a nonsteroidal anti-inflammatory drug *(increased risk of bleeding)***	Cardiovascular	Drug–drug interaction	4	3	3	95%
**Clopidogrel prescribed to a patient concomitantly with omeprazole or esomeprazole *(antiplatelet effect of clopidogrel potentially reduced)****	Cardiovascular	Drug–drug interaction	3	4	3	95%
**Macrolide antibiotic prescribed concomitantly with warfarin without appropriate dose adjustment or increased International Normalized Ratio monitoring *(increased risk of bleeding)***	Cardiovascular	Drug–drug interaction	4	4	4	95%
**Low-molecular-weight heparin omitted to be prescribed for prophylaxis *(increased risk of thrombosis)***	Cardiovascular	Omission of prophylactic treatment	4	4	4	95%
**Lithium dose not adjusted or omitted in a patient with a lithium concentration above the therapeutic range (>1.0 mmol l^{-1}) *(risk of lithium toxicity)***	Central nervous system	Dosing	4	3	3	100%
**Paracetamol prescribed at a dose of 4 g over a 24 h to a patient under 50 kg *(risk of hepatocellular toxicity)****	Central nervous system	Dosing	4	4	4	94%
**Benzodiazepine or benzodiazepine-like drug prescribed to a patient with chronic obstructive pulmonary disease *(risk of respiratory depression)***	Central nervous system	Clinical contraindication	3	3	3	90%
**Antipsychotic, other than risperidone, prescribed to a patient for the management of the behavioural and psychological symptoms of dementia *(increased risk of stroke)***	Central nervous system	Clinical contraindication	4	3	3	100%

Table 5
Continued

Prescribing safety indicator title	Group	Error type	Median scores Severity	Likelihood	Risk	Percentage agreement
**Tricyclic antidepressant prescribed to a patient with dementia *(increased risk of worsening cognitive impairment)***	Central nervous system	Clinical contraindication	3	3	3	90%
**Selective serotonin reuptake inhibitor prescribed to a patient with epilepsy *(increased risk of seizure threshold being reduced)***	Central nervous system	Clinical contraindication	3	3	3	95%
**Selective serotonin reuptake inhibitor prescribed to a patient with a history of clinically significant hyponatraemia (non-iatrogenic, sodium <130 mmol l^{-1} in the previous 2 months) *(increased risk of hyponatraemia)***	Central nervous system	Clinical contraindication	3	3	3	95%
**Prochlorperazine prescribed to a patient with parkinsonism *(risk of exacerbating parkinsonism symptoms)***	Central nervous system	Clinical contraindication	3	3	3	80%
**Nonsteroidal anti-inflammatory drug prescribed to a patient with chronic renal failure *(increased risk of deteriorating renal function)***	Central nervous system	Clinical contraindication	3	3	3	95%
**Nonsteroidal anti-inflammatory drug prescribed to a patient with a history of peptic ulcer disease or gastrointestinal bleeding without antisecretory drugs or mucosal protectants *(increased risk of peptic ulceration and bleeding)***	Central nervous system	Clinical contraindication	3	3	3	80%
**Nonsteroidal anti-inflammatory drug prescribed to a patient with a history of heart failure *(risk of exacerbation of heart failure)***	Central nervous system	Clinical contraindication	3	3	3	85%
**Lithium prescribed in conjunction with newly prescribed nonsteroidal anti-inflammatory drugs without dose adjustment or increased monitoring (*increased risk of toxicity)***	Central nervous system	Drug–drug interaction	4	3	3	100%
**Lithium therapy prescribed in conjunction with newly prescribed loop or thiazide diuretics without dose adjustment or increased monitoring (*increased risk of toxicity)***	Central nervous system	Drug–drug interaction	4	3	3	100%
**Tricyclic antidepressant prescribed at the same time as a monoamine oxidase inhibitor *(increased risk of serotonin syndrome)***	Central nervous system	Drug–drug interaction	4	2	3	80%
**Tramadol prescribed concomitantly with a monoamine oxidase inhibitor *(increased risk of serotonin syndrome)***	Central nervous system	Drug–drug interaction	4	2	3	84%
**Selective serotonin reuptake inhibitor prescribed concomitantly with tramadol *(increased risk of serotonin syndrome)***	Central nervous system	Drug–drug interaction	3	4	3	100%
**Selective serotonin reuptake inhibitor prescribed concomitantly with aspirin without appropriate prophylaxis with antisecretory drugs or mucosal protectant *(increased risk of gastrointestinal bleeding)***	Central nervous system	Drug–drug interaction	3	3	3	95%
**Citalopram prescribed concomitantly with other QT-prolonging drugs *(increased risk of arrhythmias)****	Central nervous system	Drug–drug interaction	3	3	3	85%
**Tramadol prescribed concomitantly with antiepileptics *(increased risk of seizures in patients with uncontrolled epilepsy)****	Central nervous system	Drug–drug interaction	3	4	3	100%
**Nefopam prescribed concomitantly with antiepileptics *(increased risk of seizures in patients with uncontrolled epilepsy)****	Central nervous system	Drug–drug interaction	3	3	3	80%
**Phenytoin and enteral feeds prescribed to a patient concomitantly *(reduced absorption of phenytoin)***	Central nervous system	Drug–food interaction	3	3	3	85%
**More than one paracetamol-containing product prescribed to a patient at a time *(maximal dose exceeded)***	Central nervous system	Duplicate therapy	4	3	3	95%
**Benzodiazepines prescribed long term (i.e. more than 2–4 weeks) *(risk of dependence and withdrawal reactions)***	Central nervous system	Duration	3	4	3	85%
**Benzodiazepine or benzodiazepine-like drug prescribed long term to a patient with depression *(risk of dependence and withdrawal reactions)***	Central nervous system	Duration	3	3	3	85%
**Benzodiazepine-like drugs (e.g. zopiclone) prescribed long term (i.e. more than 2–4 weeks) *(risk of dependence reactions)***	Central nervous system	Duration	3	4	3	85%
**Antipsychotic prescribed long term (i.e. >1 month) to a patient with parkinsonism *(increased risk of worsening of extrapyramidal side-effects)***	Central nervous system	Duration	3	3	3	85%
**Regular opiates prescribed without concurrent use of laxatives *(risk of severe constipation)*†**	Central nervous system	Omission of prophylactic treatment	3	4	3	85%
**Prescribing of incorrect or inequivalent morphine (opiate) dose via multiple routes *(risk of toxicity)*†**	Central nervous system	Route	3	4	3	100%
**Glibenclamide prescribed to an older adult with type 2 diabetes mellitus *(increased risk of hypoglycaemia)***	Endocrine	Clinical contraindication	3	3	3	80%
**Metformin prescribed to a patient with estimated glomerular filtration rate <30 ml min^{-1} $(1.73\ m)^{-2}$ *(increased risk of lactic acidosis)***	Endocrine	Clinical contraindication	3	3	3	90%
**Pioglitazone prescribed to a patient with heart failure *(risk of exacerbation of heart failure)****	Endocrine	Clinical contraindication	3	3	3	85%

Table 5

Continued

Prescribing safety indicator title	Group	Error type	Median scores Severity	Likelihood	Risk	Percentage agreement
**Soluble insulin prescribed to a patient on a when required basis *(increased risk of serious episodes of hypoglycaemia and nocturnal hypoglycaemia postdose)****	Endocrine	Frequency	3	3	3	90%
**Insulin prescribed to a patient at an inappropriate time, allowing for an administration without food (except once-daily long-acting insulins) *(increased risk of hypoglycaemia)***	Endocrine	Timing of dose	4	3	3	95%
**Domperidone prescribed at a total daily dose exceeding 30 mg day^{-1} in adults >60 years old *(increased risk of QTc prolongation, serious ventricular arrhythmia and sudden cardiac death)****	Gastrointestinal	Dosing	4	3	3	95%
**Diphenoxylate, loperamide or codeine phosphate prescribed as antidiarrhoeal agents for treatment of severe infective gastroenteritis (e.g. bloody diarrhoea, high fever or severe systemic toxicity) *(increased risk of exacerbation or protraction of infection)***	Gastrointestinal	Clinical contraindication	3	3	3	85%
**Metoclopramide prescribed to a patient with parkinsonism *(risk of exacerbating parkinsonism symptoms)***	Gastrointestinal	Clinical contraindication	3	4	3	85%
**Colestyramine prescribed to a patient at the same time as any other oral medication *(risk of poor clinical effect owing to reduced absorption of medications)****	Gastrointestinal	Drug–drug interaction	3	4	3	90%
**Orlistat prescribed at the same time of day as oral antiepileptics *(orlistat can reduce the absorption of antiepileptics, leading to loss of seizure control)***	Gastrointestinal	Drug–drug interaction	3	3	3	90%
**Diphenoxylate, loperamide or codeine phosphate prescribed as antidiarrhoeal agents for treatment of diarrhoea of unknown cause *(increased risk of exacerbating constipation with overflow diarrhoea)***	Gastrointestinal	Indication	3	3	3	85%
**Penicillin-containing compound prescribed to a penicillin-allergic patient without reasoning (e.g. a mild or non-allergy such as diarrhoea or vomiting entered as an allergy where the indication for penicillin is compelling) *(risk of hypersensitivity reactions)***	Infection	Allergy	4	3	3	100%
**Gentamicin dose calculated based on actual bodyweight rather than ideal bodyweight in an obese patient (body mass index >30 kg m^{-2}) *(risk of excessive dosing and toxicity)***	Infection	Dosing	4	4	4	100%
**Amphotericin B prescribed without stating the brand name and the dose in milligrams per kilogram *(risk of fatal overdose due to confusion between lipid-based and nonlipid formulations)***	Infection	Drug name	5	3	4	90%
**Cephalosporin antibiotic prescribed to an older adult (except under the direction of Microbiology or for suspected meningitis) *(increased risk of antibiotic-associated infections)***	Infection	Clinical contraindication	3	3	3	85%
**Gentamicin prescribed to a patient with renal impairment without dose adjustment *(increased risk of toxicity)***	Infection	Dosing	4	3	3	95%
**Gentamicin prescribed to an adult patient with normal renal function in a dose exceeding 7 mg kg^{-1} day^{-1} *(increased risk of toxicity)***	Infection	Dosing	4	3	3	90%
**Vancomycin prescribed intravenously to a patient with renal impairment without dose adjustment *(increased risk of toxicity)***	Infection	Clinical contraindication	4	3	3	95%
**Quinolone antibiotic prescribed to a patient with epilepsy *(increased risk of seizure threshold being reduced)***	Infection	Clinical contraindication	3	3	3	100%
**Nitrofurantoin prescribed to a patient with estimated glomerular filtration rate <60 ml min^{-1} $(1.73 m)^{-2}$ *(risk of peripheral neuropathy and inadequate concentration in urine)****	Infection	Clinical contraindication	3	3	3	90%
**Quinolone prescribed to a patient who is also receiving theophylline *(possible increased risk of convulsions)***	Infection	Drug–drug interaction	3	3	3	95%
**Atazanavir prescribed concomitantly with a proton-pump inhibitor *(concentration of atazanavir potentially reduced, reducing therapeutic effect)***	Infection	Drug–drug interaction	3	3	3	95%
**Vancomycin prescribed intravenously over less than 60 min *(rapid infusion of vancomycin can cause severe reactions)***	Infection	Intravenous rate	3	3	3	90%
**Brand specific prescribing of tacrolimus preparations *(brands vary in their dosing and pharmacokinetics)***	Miscellaneous	Drug name	4	3	3	85%
**Methotrexate prescribed to a patient with a clinically significant drop in white cell count or platelet count *(risk of bone marrow suppression)***	Miscellaneous	Clinical contraindication	4	3	3	95%
**Methotrexate prescribed to a patient with abnormal liver function tests *(risk of liver toxicity)***	Miscellaneous	Clinical contraindication	4	3	3	83%
**Potassium chloride supplements continued for longer than is required (reference range 3.5–5.3 mmol l^{-1}) *(increased risk of hyperkalaemia)***	Miscellaneous	Clinical contraindication	4	3	3	100%

Table 5
Continued

Prescribing safety indicator title	Group	Error type	Median scores Severity	Likelihood	Risk	Percentage agreement
**Methotrexate prescribed concomitantly with trimethoprim *(increased risk of haematological toxicity)***	Miscellaneous	Drug–drug interaction	3	3	3	89%
**Weekly dose of an oral bisphosphonate prescribed daily *(risk of hypocalcaemia)****	Miscellaneous	Frequency	3	3	3	89%
**Oral methotrexate prescribed to a patient with an inappropriate frequency *(increased risk of toxicity)***	Miscellaneous	Frequency	5	2	3	89%

*Additional indicators recommended by panellists in the exploratory round and included in round one ($n = 13$). †Indicators where the text was suggested to be modified by panellists in the exploratory round for round one ($n = 2$). Median risk scores are as follows: 1, low risk; 2, moderate risk; 3, high risk; and 4, extreme risk.

The 80 indicators included a total of 41 different trigger drugs or classes, prescribed for the following indications: cardiovascular ($n = 22$), central nervous system ($n = 28$), endocrine ($n = 5$), gastrointestinal ($n = 6$), infection ($n = 12$) and miscellaneous ($n = 7$). The most common drugs and drug classes named in the indicators were antibiotics ($n = 13$), antidepressants ($n = 8$), nonsteroidal anti-inflammatory drugs ($n = 6$), opioid analgesics ($n = 6$), antiplatelet medications ($n = 5$), methotrexate ($n = 4$), low-molecular-weight heparins ($n = 4$), and benzodiazepine (and benzodiazepine-like) drugs ($n = 4$).

Participants identified five indicators as 'extreme risk', calculated using the NPSA Risk Matrix. Three of these involved anti-infective drugs [macrolides (with warfarin), gentamicin and amphotericin B], one involved a low-molecular-weight heparin and one related to paracetamol. The most frequent error types identified as high or extreme risk were those classified as clinical contraindications ($n = 29$ of 80). This included drugs prescribed in renal impairment ($n = 8$), heart failure ($n = 4$) and epilepsy ($n = 4$), as well as those that should be avoided with abnormal blood results ($n = 4$). Drug–drug interactions were the second most common error type ($n = 23$ of 80), with antidepressants being the most common interacting drug class ($n = 5$).

When indicators were ranked according to median severity scores only, only two indicators were given a score of '5' (catastrophic; see Table 2), as follows: amphotericin B prescribed without stating the brand name and the dose in milligrams per kilogram (risk of fatal overdose due to confusion between lipid-based and nonlipid formulations); and oral methotrexate prescribed to a patient with an inappropriate frequency (increased risk of toxicity).

Discussion

This eDelphi has identified 80 high- and extreme-risk prescribing indicators that are relevant to the hospital setting, which also have the potential to be prevented by alerts and warnings in decision support software. All 20 participants completed the exploratory round and both rounds of the eDelphi, removing any bias potentially introduced by missing responses from people with specific expertise.

The most frequently named drugs in the final list are antibiotics, opioids and low-molecular-weight heparins. This is consistent with the drugs and drug classes considered high risk by the NPSA, identified in incident reports with clinical outcomes of death and severe harm [23]. Opioid analgesics, antibiotics, warfarin and low-molecular-weight heparins are the drugs with the highest percentage of medication incident reports with fatal and severe harm outcomes. Indicators relating to antidepressants were also frequent, but are not listed by the NPSA as drugs with a high number of reports for fatal and severe harm. Four of these were for selective serotonin reuptake inhibitors, a class of drug with a high prescribing rate in UK, with citalopram being one of the top 20 drugs dispensed by pharmacies in England [24].

The most frequent error types associated with the indicators were clinical contraindications ($n = 29$ of 80) and drug–drug interactions ($n = 23$ of 80). In the USA, a set of high-priority drug–drug interactions were developed by an expert panel to help target CDS and create a list of interactions as a minimal standard for such systems [17, 25]. Interestingly, only six of the 23 drug–drug interactions identified by the expert panel in this eDelphi process were the same as those previously identified by Phansalkar *et al.* [17], showing there to be a difference in opinion between what the UK and the USA would consider to be highly significant. This may, in part, be due to the difference in the rate at which these drugs are prescribed in each country. However, when the scoring for the indicators was ranked according to median severity scores only, two indicators scored '5' (catastrophic) and 26 scored '4' (major); nine of these were drug–drug interactions, and only one was consistent with the list defined by Phansalkar *et al.* [17].

The prescribing indicators were developed for the hospital setting and therefore include some drugs which are not likely to be prescribed in general practice (e.g.

Table 6

Indicators not considered high or extreme risk by consensus of at least 80%

Prescribing safety indicator title
Proton-pump inhibitors prescribed at the same time as antacid formulations *(reduced therapeutic effect of the proton-pump inhibitor)*
Thiazide diuretic prescribed to a patient with a history of gout *(increased risk of exacerbating symptoms in pre-existing gout)*
Thiazide prescribed to a patient with chronic kidney disease stage 3 (estimated glomerular filtration rate <45 ml^{-1} min^{-1} (1.73 $m)^{-2}$) or above *(increased risk of adverse effects)*
β-Adrenoceptor-blocking drug prescribed to a patient with asthma *(increased risk of bronchospasm and acute deterioration)*
Aliskiren prescribed concomitantly with angiotensin-converting enzyme inhibitors or angiotensin II receptor antagonists *(increased risk of serious adverse cardiovascular and renal outcomes)*
Aliskiren prescribed to a patient with severe renal impairment, estimated glomerular filtration rate <30 ml min^{-1} (1.73 $m)^{-2}$ *(risk of hyperkalaemia)*
Long-acting inhaled antimuscarinic prescribed concomitantly with a short-acting nebulized antimuscarinic *(increased risk of additive adverse effects)*
Selective serotonin reuptake inhibitor prescribed concomitantly with pethidine *(increased risk of serotonin syndrome)*
Selective serotonin reuptake inhibitors prescribed at the same time as monoamine oxidase inhibitors *(increased risk of serotonin syndrome)*
Metoclopramide prescribed to a patient <20 years (except in cases of severe intractable vomiting of known cause, or due to cytotoxics/radiotherapy) (*increased risk of extrapyramidal side-effects)*
Two concomitant opiate analgesics that are not in line with the World Health Organization pain ladder *(injudicious use of two opiates)*
Aspirin prescribed to a child ≤16 years (except in Kawasaki's disease) *(increased risk of Reye's syndrome)*
Vancomycin prescribed intravenously for the treatment of *Clostridium difficile* infection *(intravenous vancomycin has limited therapeutic effect)*
Oral quinolone antibacterial prescribed at the same time as iron *(reduced absorption of quinolones)*
Triazole antifungal prescribed at the same time as fentanyl *(increased risk of opiate toxicity)*
Rifampicin prescribed concomitantly with ritonavir *(ritonavir concentration can be reduced, reducing its effect)*
Bisphosphonate prescribed to a patient with an inappropriate timing *(increased risk of adverse effects and possible reduced absorption if given after food)*
Bisphosphonate prescribed to a patient at the same time of day as calcium *(bisphosphonate absorption reduced by calcium salts)*
Methotrexate prescribed on the same day as folic acid *(reduced efficacy of methotrexate)*
Allopurinol prescribed concomitantly with azathioprine *(allopurinol enhances effect of azathioprine and increases risk of toxicity)*
Allopurinol prescribed concomitantly with mercaptopurine *(allopurinol enhances effect of mercaptopurine and increases risk of toxicity)*
Calcium resonium prescribed when the potassium concentration is within the desired reference range (3.5–5.3 mmol l^{-1}) *(risk of hypokalaemia)*
Potassium chloride infusions exceeding 40 mmol l^{-1} given via the peripheral route *(peripheral administration risks venous pooling, which can lead to sudden high concentrations of potassium chloride being delivered to the heart, provoking an arrhythmia)*
Selective cyclo-oxygenase-2 inhibitor nonsteroidal anti-inflammatory drug prescribed to a patient with cardiovascular disease *(increased risk of thrombotic events)*
More than one nonsteroidal anti-inflammatory drug prescribed to a patient at a time *(increased risk of bleeding)*
Allopurinol prescribed at a dose exceeding 100 mg in a patient with renal impairment *(risk of accumulation and subsequent toxicity)*
Live vaccine prescribed to an immunosuppressed patient, including those on corticosteroids *(increased risk of reaction or infection)*
Two loop diuretics prescribed concomitantly *(increased risk of adverse effects)*
Long-acting β_2-agonist inhaler prescribed to a patient who is not also on an inhaled corticosteroid *(evidence base – not in line with British Thoracic Society guidelines)*

intravenous gentamicin). However, only six such indicators out of the 80 include drugs which would be prescribed only on an in-patient basis, and therefore 74 of the indicators have the potential to be applied to general practice. Indeed, eight of the final indicators were taken directly from Avery *et al*. [16], which were originally developed for GP systems, and were subsequently scored as high or extreme risk in this eDelphi process.

The indicators of harm identified provide an objective measure that can be implemented in the routine data collection of prescribing errors in both paper-based and electronic processes. The indicators can be identified during prospective prescription chart review, and their presence documented. Following the implementation of a safety improvement programme or intervention, it would be intended that the same prescription review process would be carried out (i.e. 'pre/post' studies). The collection of standardized data in this way allows for comparison to be made and conclusions drawn which can provide evidence for safety initiatives. With the capital cost of installing a CPOE system in a hospital being in the region of £1.5 million, research into its effect and effectiveness are crucially important. Investigating the rate of prescribing errors pre- and post implementation in such cases is beneficial in providing evidence to support one of the primary objectives of implementing such a system, i.e. to reduce the number of medication errors and subsequent harms.

The indicators are not restricted to one type of error, unlike similar studies [17], and therefore include error types such as 'dosing', which represent one of the largest error categories in the UK [23, 26]. The ability to capture a broad range of errors allows for more than one type of safety improvement strategy to be tested. Furthermore, the use of a standard data collection tool between hospital sites allows for valid comparisons to be made.

Finally, as a secondary outcome of this research, the indicators identified could be used to inform the development of decision support warnings or alerts, with the intention of minimizing untargeted or nonspecific alerting, which can lead to an overburdening of the prescriber and cause 'alert fatigue', limiting its intended effects [27, 28]. A Cochrane review in 2011 found that point-of-care computer reminders generally achieve small-to-modest improvements in provider behaviour [29], and concluded that further research must identify key factors – related to the 'target quality' problem or the design of the reminder – that reliably predict larger improvements in care from such expensive technologies. The indicators developed here can help to ensure that CDS targets the errors that are more likely to occur and/or have the greatest potential for causing patient harm, and may serve as a priority list for CDS software developers.

Limitations

Prior to the commencement of the eDelphi, 210 indicators were identified by the research team from both clinical experience and published literature. Of these indicators, 130 were excluded by the same members of the team because it was felt that they were neither prescribed at a reasonable frequency nor considered to be sufficiently high risk for inclusion. This review process meant that many of the indicators sent to the participants could already be considered high risk, and may explain why the final list was not substantially smaller. The same 20 participants also took part in both the exploratory round and the two-round eDelphi, which may further explain why consensus was reached on a large number of indicators. We acknowledge that there may also be a risk that in the original identification step we missed some high-risk errors, despite a robust review of the literature, or excluded some that other people would have considered important enough for inclusion. However, the exploratory round prior to the eDelphi process was designed to reduce the risk of such omissions in the final list.

All participants in the eDelphi were from geographically diverse areas in England. However, the lack of expertise from further afield may make this tool more specifically applicable to the UK setting. Indeed, it may be of interest to see whether, for example, experts from other defined geographical regions (e.g. USA and other European countries) would come to similar conclusions as UK healthcare professionals.

Finally, with the development of any indicator or trigger to monitor quality or safety in healthcare, its relevance should be continuously reviewed and updated. As new therapeutic agents are introduced and older ones go out of favour, the likelihood scores for their occurrence in clinical practice may well adjust, and they would no longer qualify according to our methodology.

Conclusions

Prescribing errors with high potential for causing patient harm have been identified by an expert panel. These indicators provide a standardized, validated tool for the routine collection of prescribing error data in both paper-based and electronic prescribing processes. They can serve as a means to assess safety improvement programmes pre- and post implementation, such as with the introduction of CPOE and CDS. This tool could also be of value in the refinement of alerts and warnings embedded within CPOE systems to ensure that they are targeted and that the alert burden on physicians is rationalized.

Competing Interests

All authors have completed the Unified Competing Interest form at http://www.icmje.org/coi_disclosure.pdf (available on request from the corresponding author) and declare: S.K.T., S.E.McD., J.H., U.N., R.L.H., A.J.A. and J.J.C. had financial support in the form of a research grant from the National Institute for Health Research (NIHR) for the

submitted work; no financial relationships with any organizations that might have an interest in the submitted work in the previous 3 years; no other relationships or activities that could appear to have influenced the submitted work.

The authors wish to thank the members of the expert panel for their contributions to this study. This work is funded by the National Institute for Health Research (NIHR). S.K.T., S.E.McD., J.H. and U.N. are funded through the Collaborations for Leadership in Applied Health Research and Care for Birmingham and Black Country (CLAHRC-BBC) programme. J.J.C., R.L.H. and A.J.A. are funded by a National Institute for Health Research (NIHR) programme grant: 'Investigating the implementation, adoption and effectiveness of ePrescribing systems in English hospitals'.

The views expressed in this publication are not necessarily those of the NIHR, the Department of Health, NHS Partner Trusts, Universities of Birmingham, Nottingham or Reading, CLAHRC-BBC Theme 9 Management/Steering Group.

REFERENCES

1 Davies EC, Green CF, Taylor S, Williamson PR, Mottram DR, Pirmohamed M. Adverse drug reactions in hospital in-patients: a prospective analysis of 3695 patient-episodes. PLoS ONE 2009; 4: e4439.

2 Dean B, Barber N. A validated, reliable method of scoring the severity of medication errors. Am J Health Syst Pharm 1999; 56: 57–62.

3 Dornan T, Ashcroft D, Heathfield H, Lewis P, Taylor D, Tully MP, Wass V. An in depth investigation into causes of prescribing errors by foundation trainees in relation to their medical education. EQUIP study. 2009.

4 Avery T, Barber N, Ghaleb MA, Dean Franklin B, Armstrong S, Crowe S, Dhillon S, Freyer A, Howard R, Pezzolesi C, Serumaga B, Swanwick G, Talabi O. Investigating the prevalence and causes of prescribing errors in general practice: the PRACtICe Study. A report for the GMC: General Medical Council, 2012.

5 Griffin FA, Resar RK. IHI Global Trigger Tool for Measuring Adverse Events (Second Edition). *IHI Innovation Series White Paper.* Cambridge, MA: Institute for Healthcare Improvement, 2009.

6 The Information Centre (Health Care). The Prescribing toolkit. Available at http://www.ic.nhs.uk/prescribing/measures (last accessed 22 August 2012).

7 Avery AJ, Dex GM, Mulvaney C, Serumaga B, Spencer R, Lester HE, Campbell SM. Development of prescribing-safety indicators for GPs using the RAND Appropriateness Method. Br J Gen Pract 2011; 61: e526–36.

8 Gallagher P, Ryan C, Byrne S, Kennedy J, O'Mahony D. STOPP (Screening Tool of Older Person's Prescriptions) and START (Screening Tool to Alert doctors to Right Treatment). Consensus validation. Int J Clin Pharmacol Ther 2008; 46: 72–83.

9 Laroche ML, Charmes JP, Merle L. Potentially inappropriate medications in the elderly: a French consensus panel list. Eur J Clin Pharmacol 2007; 63: 725–31.

10 NSW Therapeutic Advisory Group. Indicators for quality use of medicines in Australian hospitals. Available at http://www.ciap.health.nsw.gov.au/nswtag/reviews/indicators.html (last accessed 3 January 2013).

11 Schedlbauer A, Prasad V, Mulvaney C, Phansalkar S, Stanton W, Bates DW, Avery AJ. What evidence supports the use of computerized alerts and prompts to improve clinicians' prescribing behavior? J Am Med Inform Assoc 2009; 16: 531–38.

12 Kaushal R, Shojania KG, Bates DW. Effects of computerized physician order entry and clinical decision support systems on medication safety: a systematic review. Arch Intern Med 2003; 163: 1409–16.

13 Pearson SA, Moxey A, Robertson J, Hains I, Williamson M, Reeve J, Newby D. Do computerised clinical decision support systems for prescribing change practice? A systematic review of the literature (1990–2007). BMC Health Serv Res 2009; 9: 154.

14 Avery AJ, Savelyich BSP, Sheikh A, Cantrill J, Morris CJ, Fernando B, Bainbridge M, Horsfield P, Teasdale S. Identifying and establishing consensus on the most important safety features of GP computer systems: e-Delphi study. Inform Prim Care 2005; 13: 3–12.

15 NIHR Programme Grant for Applied Research. E-prescribing: everything you want to know but were afraid to ask! A symposium for the health service. Available at http://www.cphs.mvm.ed.ac.uk/projects/eprescribing/index.html (last accessed 11 November 2012).

16 Avery AJ, Dex GM, Mulvaney C, Serumaga B, Spencer R, Lester HE, Campbell SM. Development of prescribing-safety indicators for GPs using the RAND Appropriateness Method. Br J Gen Pract 2011; 61: e526–e36.

17 Phansalkar S, Desai AA, Bell D, Yoshida E, Doole J, Czochanski M, Middleton B, Bates DW. High-priority drug–drug interactions for use in electronic health records. J Am Med Inform Assoc 2012; 19: 735–43.

18 British Medical Association and the Royal Pharmaceutical Society of Great Britain. British National Formulary. Available at http://www.bnf.org (last accessed 1 March 2012).

19 National Patient Safety Agency. Available at http://www.npsa.nhs.uk (last accessed 1 March 2012).

20 Medicines and Healthcare products Regulatory Agency. Available at http://www.mhra.gov.uk (last accessed 1 March 2012).

21 National Patient Safety Agency. A risk matrix for risk managers, 2008.

22 Fitch K, Bernstein SJ, Aguilar MS, Burnand B, LaCalle JR, Lazaro P, van het Loo M, Mcdonnell J, Vader J, Kahan JP. The RAND/UCLA Appropriateness Method User's Manual. Santa Monica, CA: RAND, 2001.

23 Cousins DH, Gerrett D, Warner B. A review of medication incidents reported to the National Reporting and Learning

System in England and Wales over six years (2005–2010). Br J Clin Pharmacol 2011; 74: 597–604.

24 The NHS Health and Social Care Information Centre. Prescriptions dispensed in the community: England, statistics for 2001 to 2011, 2012.

25 Classen D, Phansalkar S, Bates D. Critical drug-drug interactions for use in electronic health records systems with computerized physician order entry: review of leading approaches. J Patient Saf 2011; 7: 61–5.

26 National Patient Safety Agency. Safety in doses: improving the use of medicines in the NHS. 2008.

27 van der Sijs H, Aarts J, van Gelder T, Berg M, Vulto A. Turning off frequently overridden drug alerts: limited opportunities for doing it safely. J Am Med Inform Assoc 2008; 15: 439–48.

28 Riedmann D, Jung M, Hackl WO, Stuhlinger W, van der Sijs H, Ammenwerth E. Development of a context model to prioritize drug safety alerts in CPOE systems. BMC Med Inform Decis Mak 2011; 11: 35.

29 Shojania KG, Jennings A, Mayhew A, Ramsay CR, Eccles MP, Grimshaw J. The effects of on-screen, point of care computer reminders on processes and outcomes of care. Cochrane Database Syst Rev 2009; (3): CD001096.
